# Variability of internal and external loads and technical/tactical outcomes during small-sided soccer games: a systematic review

**DOI:** 10.5114/biolsport.2022.107016

**Published:** 2021-08-27

**Authors:** Filipe Manuel Clemente, Rodrigo Aquino, Gibson Moreira Praça, Markel Rico-González, Rafael Oliveira, Ana Filipa Silva, Hugo Sarmento, José Afonso

**Affiliations:** 1 Escola Superior Desporto e Lazer, Instituto Politécnico de Viana do Castelo, Rua Escola Industrial e Comercial de Nun’Álvares, 4900-347 Viana do Castelo, Portugal; 2 Instituto de Telecomunicações, Delegação da Covilhã, Lisboa 1049-001, Portugal; 3 LabSport, Department of Sports, Center of Physical Education and Sports, Federal University of Espírito Santo, Vitória, Espírito Santo, Brazil; 4 Sports Department, Universidade Federal de Minas Gerais, Belo Horizonte, Brazil; 5 Department of Physical Education and Sport, University of the Basque Country, UPV-EHU, Lasarte 71, 01007 Vitoria-Gasteiz, Spain; 6 Sports Science School of Rio Maior–Polytechnic Institute of Santarém, 2140-413, Rio Maior, Portugal; 7 Life Quality Research Centre, 2140-413, Rio Maior, Portugal; 8 N2i, Polytechnic Institute of Maia, 4475-690 Maia, Portugal; 9 The Research Centre in Sports Sciences, Health Sciences and Human Development (CIDESD), Vila Real 5001-801, Portugal; 10 University of Coimbra, Research Unit for Sport and Physical Activity. Faculty of Sport Sciences and Physical Education, Coimbra, Portugal; 11 Centre for Research, Education, Innovation and Intervention in Sport, Faculty of Sport of the University of Porto, Porto, Portugal

**Keywords:** Football, Performance, Athletic performance, Conditioned games, Training load, Motor skills

## Abstract

Small-sided games (SSGs) are widely used in soccer training. However, some of the typical outcomes related to human responses during these games (namely internal and external load) may vary between sessions for similar practice conditions. Thus, the study of intra- and inter-bout variability in response to SSGs is progressively growing. This systematic review aimed to (1) identify studies that have examined the intra- and inter-session bouts’ variability levels regarding the internal and external load and technical/tactical outcomes during SSGs and (2) summarize the main evidence. A systematic review of PubMed, SPORTDiscus, Cochrane, and Web of Science databases was performed according to the Preferred Reporting Items for Systematic Reviews and Meta-Analyses (PRISMA) guidelines. From the 486 studies initially identified, 24 were fully reviewed, and their outcome measures were extracted and analyzed. Sixteen studies analyzed internal load, 13 studies analyzed external load variables, six studies analyzed technical execution, and two studies analyzed tactical behavior. All studies included SSGs with a range number of players between 2 to 14 (1 vs. 1 to 7 vs. 7 SSGs). Internal load and low-speed external load variables presented a low variability, while high variations were reported regarding the technical execution and high-speed external loads.

## INTRODUCTION

Small-sided games (SSGs) are conditioned forms of official games in which specific task constraints are adjusted to promote new challenges in a tactical/technical dimension [1]. Such adjustments promote variations in physiological and physical demands [2]. These drill-based tasks are very popular in soccer since they seek to promote specificity of practice reflecting the dynamics of the game [3]. In fact, some training protocols use SSGs for promoting physical development in players [4]. However, since they are drill-based games—and due to the proper dynamics of the match—it is expectable that these games promote a considerable intra- and inter-player variability in their acute responses to exercise [5].

There is an extensive body of knowledge focused on the acute and chronic responses promoted by SSGs in physiological and physical demands [6, 7], as well as a large body of knowledge about its effects on tactical and technical dimensions [3, 8]. Briefly, acute responses traditionally cover the impact of SSGs on internal load (the physiological responses to a given physical demand imposed by the exercise/drill) and external load (the physical demands imposed by the exercise/drill) [9]. They also encompass SSGs’ effects on technical responses (technical actions and their accuracy performed during the games) and tactical behaviors (individual behaviors related to the dynamics of the game and interactions with teammates, opponents, and the ball) [10]. In fact, it is expectable a close relationship between all the above-mentioned outcomes (load, technical and tactical) namely because SSGs reproduce the dynamic of the formal match. In that sense, it is expectable that the emergent behaviors and collective and individual dynamics will change the external load of the match (e.g., influence by contextual factors) [11, 12]. Such fact will promote natural consequences in the physiological responses since the well-reported relationships between some internal and external load measures [13].

In studies conducted on SSGs among soccer players, heart rate and rate of perceived exertion are the outcomes most commonly related to internal load [7, 14, 15]. However, in some cases, blood lactate concentration is also reported. The intra- and inter-player variability for these outcomes have been studied during SSGs, with findings suggesting that heart rate responses present lower variability [16–18], while blood lactate concentrations and perceived exertion are more variable [16, 19].

In the case of external load, total distance, distances covered at high demands—for instance, high-speed running (> 19.8 km/h) or sprinting (> 25 km/h)—and the number of accelerations or decelerations are the most frequent outcomes presented by original articles [6, 7]. Some studies have revealed that among these outcomes, total distance has relatively low intra- and inter-player variability during SSGs [20, 21], while distances covered at high-intensity have relatively high variability [17, 20, 21].

Regarding technical actions, passes, shots, and receptions are some of the most commonly reported outcomes [8]. Regarding tactical behaviors, some principles of play related to attacking or defending, as well as exploratory behaviors related to playing position in the Cartesian space, are often presented in the literature [3, 22]. Despite the small number of studies analyzing the variability of technical and tactical responses during SSGs compared to internal and external load demands, the reports suggest more variability among technical and tactical outcomes [18, 23].

As presented above, studies on the within- and between-session variability of internal and external load and technical/tactical dimensions during the same SSGs have become prominent in recent years [16, 17, 20]. However, as far we know, no systematic review has summarized the evidence about this kind of variability across different original studies. A summary of the variability levels of SSGs (intra-SSGs variability) may provide information vital to identifying the impact of these games on different outcomes and select the most appropriate games and formats to apply to aim to ensure a proper stimulus in specific outcomes. Thus, coaches may decide to use SSGs to work on some variables and other training methods to work on others.

Therefore, the first purpose of this systematic review is to identify studies that have examined the impact of intra- and inter-SSG bouts/sets on soccer players’ variability levels of internal and external load and technical/tactical outcomes. The second purpose is to summarize the main evidence presented in the literature. However, in some cases, the specific instruments may be the cause for the variability, and so special attention will be given to that fact during the synthesis of results of the current systematic review.

## MATERIALS AND METHODS

The systematic review strategy was conducted according to PRISMA (Preferred Reporting Items for Systematic Reviews and Meta-analyses) guidelines [24]. The P.I.C.O.S. (Population or problem; Intervention or exposure; Comparison; Outcome; Study design) was established (Table 1). The protocol was registered with the International Platform of Registered Systematic Review and Meta-Analysis Protocols with the number 202130080 and the DOI number INPLASY202130080.

**TABLE 1:** Inclusion and exclusion criteria.

	Inclusion criteria	Exclusion criteria
Population	Soccer players of any age or sex, with regular training practice and without major injury or illness.	Sports other than soccer (e.g., rugby, American football, handball, volleyball, futsal, basketball) Players with major injuries or illness.
Intervention	A minimum of two bouts/sets of a SSG (within- or between-sessions). Thus, the same game was made at least twice in a single session or at least one time in two different training sessions. AND The exact same conditions of practice (e.g., same teams, same format of play) were made between repetitions	SSGs with single bout/set; The bouts/sets changes the constraints (e.g., play format, court dimensions) or conditions (change teams and players within or between-sessions); The conditions changes by any exercise or test (e.g., inducing mental or physical fatigue) made between repetitions occurring in the same session
Comparator	The comparators are the different bouts/sets of each SSG.	Single bout/set of an SSG.
Outcome	Any measure of variability (e.g., ICC, CV, etc.) or any metrics that, combined, afford calculation of variability (e.g., mean ± SD, mean ± SEM). One of the following outcomes should be included: Internal load [heart rate; blood lactate concentrations; rate of perceived exertion]; External load [total distance; distances between 19.8 and 25 km/h; distances > 25 km/h; accelerations and decelerations]; Technical actions [passes; receptions; ball touches; shots]; Tactical behavior [attacking behaviors; defensive behaviors]	Does not present a measure of variability (e.g., ICC, CV, etc.) or any metrics that, combined, afford calculation of variability (e.g., mean ± SD, mean ± SEM). Does not present at least one of the following outcomes. Examples of exclusion: Well-being parameters related to measures of fatigue, stress, mood, recovery, sleep quality or others; Psychological or sociological outcomes as enjoyment or cohesion; Readiness parameters as heart rate variability, neuromuscular capacity or others.
Study design	Repeated measures design with the same players and teams	No repeated measures design with the same players and teams Experimental studies analyzing the effects of SSGs training protocols on fitness/technical or tactical variables
Additional criteria	Original research published in peer-review journals, restricted to English, Portuguese and Spanish and no limited to date.	Written in other language than English, Portuguese or Spanish. Other article types than original (e.g., reviews, letters to editors, trial registrations, proposals for protocols, editorials, book chapters and conference abstracts).

### Eligibility criteria

The inclusion and exclusion criteria can be found in table 1.

The screening of the title, abstract and reference list of each study to locate potentially relevant studies was independently performed by the two authors (MRG and JA). Additionally, they reviewed the full version of the included papers in detail to identify articles that met the selection criteria. An additional search within the reference lists of the included records was conducted to retrieve additional relevant studies. A discussion was made in the cases of discrepancies regarding the selection process with a third author (HS). Possible errata for the included articles were considered.

### Information sources and search

Electronic databases (PubMed, SPORTDiscus, Cochrane and Web of Science – core collection) were searched for relevant publications prior to the February 9 of 2021. Keywords and synonyms were entered in various combinations: title (i.e., “Soccer” OR “Football”) AND title (“small-sided” OR “SSG” OR “conditioned”) AND in the title, abstract or keywords (“varia*” OR “reproducibility” OR “repeatability” OR “reliability”). Additionally, the reference lists of the studies retrieved were manually searched to identify potentially eligible studies not captured by the electronic searches. Finally, an external expert has been contacted in order to verify the final list of references included in this scoping review in order to understand if there was any study that was not detected through our research. Possible errata was searched for each included study.

### Data Extraction

A data extraction was prepared in Microsoft Excel sheet (Microsoft Corporation, Readmon, WA, USA) in accordance with the Cochrane Consumers and Communication Review Group’s data extraction template [25]. The Excel sheet was used to assess inclusion requirements and subsequently tested for all selected studies. The process was independently conducted by two of the authors (FMC and HS). Any disagreement regarding study eligibility was resolved by a third author (JA). Full text articles excluded, with reasons, were recorded. All the records were stored in the sheet.

### Data items

The intraclass correlation coefficient (ICC) and/or typical error of measurement (TEM) (%) and/or coefficient of variation (CV) (%) and/or standard error of measurement (SEM) values were extracted from the original articles regarding the following measures: (i) internal load [e.g., heart rate; blood lactate concentrations; rate of perceived exertion]; (ii) external load [e.g., total distance; distances between 19.8 and 25 km/h; distances > 25 km/h; accelerations and decelerations]; (iii) technical actions [e.g., passes; receptions; ball touches; shots]; and (iv) tactical behavior [e.g., attacking behaviors; defensive behaviors]. Additionally, the following information was extracted from the included studies: (i) number of participants (n), age (years), competitive level (if available) and sex; (ii) the SSGs format, pitch configuration and other information about tactical conditions; (iii) number of repetitions and sessions analyzed; (iv) training regimen (work duration, work intensity, modality, relief duration, relief intensity, repetitions and series, between-set recovery); and (v) instruments used to monitor the load and the errors reported to those instruments.

### Assessment of methodological quality

Adapted version of STROBE assessment was used to evaluate the included articles´ eligibility [26]. Any disagreement was discussed and solved by consensus decision. Each of ten items was qualified using numerical codification (1 = considered or 2 = non-considered). Those studies with more than 7 complete items (score of 7 is not included), are considered as a low risk of bias.

## RESULTS

### Study identification and selection

**FIG. 1:**
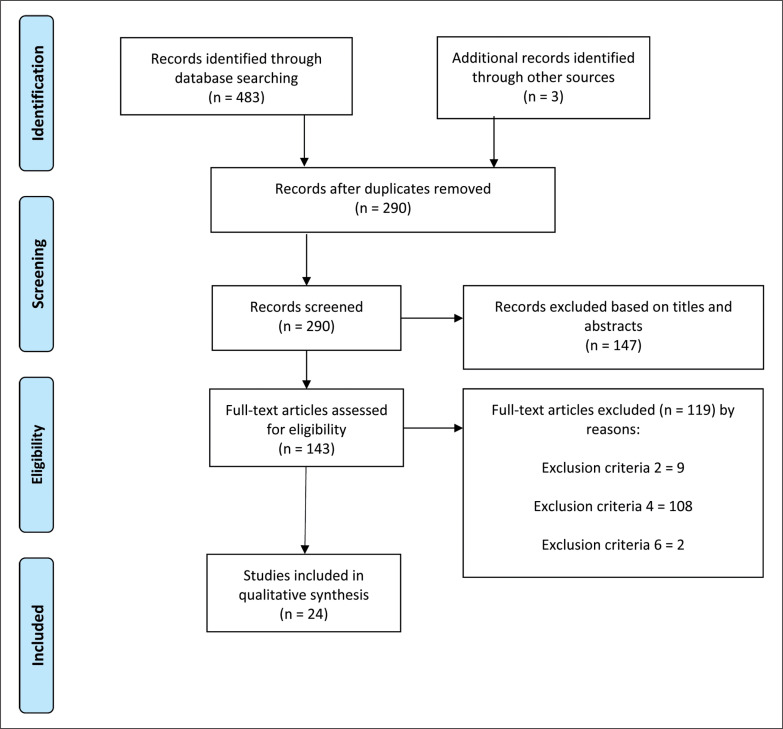
PRISMA flow diagram highlighting the selection process for the studies included in the current systematic review.

The searching of databases identified an initial 483 titles, and 3 was found from external sources. These studies were then exported to reference manager software (EndNote^TM^ X9, Clarivate Analytics, Philadelphia, PA, USA). Duplicates (196 references) were subsequently removed either automatically or manually. The remaining 290 articles were screened for their relevance based on titles and abstracts, resulting in the removal of a further 147 studies. Following the screening procedure, 143 articles were selected for in depth reading and analysis. After reading full texts, a further 119 studies were excluded owing to a number of reasons including: exclusion criteria 2, 4, and 6. Therefore, 24 articles were eligible for the systematic review (Figure 1). The twenty-four studies included provided mean and standard deviation of reliability data.

### Methodological quality

The table 2 presents the summary of methodological assessment. From the 24 included articles, nine were classified as low methodological quality (37.5%), while the remaining were classified as high-quality.

**TABLE 2:** Methodological assessment.

Study	1	2	3	4	5	6	7	8	9	10	Quality
[27]	1	1	0	1	1	1	1	1	1	0	High
[49]	1	1	1	1	1	1	1	1	1	0	High
[53]	1	1	1	1	1	1	1	1	1	0	High
[54]	1	0	0	1	1	1	1	1	1	1	High
[55]	1	1	1	1	1	1	1	1	1	0	High
[29]	1	1	1	1	1	1	1	1	1	1	High
[30]	1	1	0	1	1	1	1	1	1	1	High
[45]	1	0	0	1	1	1	1	1	1	0	Low
[56]	1	0	1	1	1	1	1	1	1	1	High
[34]	1	1	1	1	1	1	1	0	0	1	High
[37]	1	1	1	1	1	1	1	0	0	1	High
[57]	1	1	0	1	1	1	1	0	0	1	Low
[58]	1	1	0	1	1	1	1	0	1	1	High
[48]	1	1	0	1	1	1	1	0	0	0	Low
[16]	0	0	0	1	1	1	1	0	0	0	Low
[59]	1	0	1	1	1	1	1	1	0	1	High
[35]	1	0	1	1	1	1	1	0	0	1	Low
[46]	1	0	0	1	1	1	1	1	1	1	High
[36]	1	1	1	1	1	1	1	1	1	0	High
[60]	1	0	1	1	1	1	1	0	0	0	Low
[31]	1	0	0	1	1	1	1	0	0	1	Low
[61]	1	0	0	1	1	1	1	0	0	0	Low
[62]	1	0	0	1	1	1	1	1	1	0	Low
[21]	1	1	1	1	1	1	1	1	1	1	High

**Note:** provide in the abstract an informative and balanced summary of what was done and what was found (item 1); state specific objectives, including any prespecified hypotheses (item 2); Give the eligibility criteria, and the sources and methods of selection of participants (item 3); for each variable of interest, give sources of data and details of methods of assessment (measurement). Describe comparability of assessment methods if there is more than one group (item 4); explain how quantitative variables were handled in the analyses. If applicable, describe which groupings were chosen and why (item 5); give characteristics of study participants (item 6); summarize key results with reference to study objectives (item 7); discuss limitations of the study, considering sources of potential bias or imprecision. Discuss both direction and magnitude of any potential bias (item 8); give a cautious overall interpretation of results considering objectives, limitations, multiplicity of analyses, results from similar studies, and other relevant evidence (item 9); give the source of funding and the role of the funders for the present study and, if applicable, for the original study on which the present article is based (item 10).

### Study characteristics

The characteristics of the studies included in the systematic review can be found in table 3. From the total number of 24 studies, 16 studies analysed internal load, 13 studies analysed external load variables, six studies analysed technical execution and two studies analysed tactical behavior.

**TABLE 3:** Study characteristics.

Study	Formats	N | Age | CL	IL and EL instruments and error	TE and TB instruments and error	IL outcomes	EL outcomes	TE outcomes	TB outcomes
[27]	4 vs 4 Within session	N = 16 Age:23.9 ± 4.2 CL:A	IL: RPE (CR-20 scale) HR (Polar Team Sport System, Polar Electro Oy, Finland EL: Ultra-Wide Band (WIMU PROTM) and PROTM software (RealTrack Systems, Almeria, Spain)	None	RPE (CR-20 scale) HRpeak HRAvg % HRpeak < 75% HRpeak 75–84% HRpeak 85–89% HRpeak > 90% HRpeak	TD Distances at 0.1–6.9 7.0–12.9 13.0–17.9 ≥ 18.0 km·h^-1^ Total m/min Total Acc nr Acc (1.0–1.4) Acc (1.5–1.9) Acc (2.0–2.4) Acc (≥ 2.5 m·min^-2^) TDec nr Max speed Avg speed	None	None
[49]	3 vs 3 4 vs 3 Within session	N = 18 Age: 16.4 ± 0.4 CL: A	IL: HR (Polar®, FS1 transmitters, Finland) Standard error of measurement: HRAvg = 8.9–6.7% HRpeak = 5.6–6.0% EL: 15 Hz GPS (GPSports Systems model SPIProX2) Standard error of measurement: TD = 22.6–29.5% D% 0–7.2 km = 11.2–13.2% D% 7.3–14.3 km = 22.1–24.0% D% 14.4–21.5 km = 17.3–21.3% Max speed = 2.3–2.4% Acc nr (> 2 m/s) = 1.4–2.1% % D Acc (> 2 m/s) = 21.3–22.5% Acc max = 0.4–0.4%	TB: Soccer Analyser® software and System of Tactical Assessment in Soccer (FUT-SAT) with Kappa coefficient values above 0.9 Standard error of measurement: Penetration = 1.9–1.9% Offensive coverage = 2.0–2.8% Width and length with ball = 1.1–1.0% Width and length without ball = 3.3–2.9% Depth mobility = 1.4–2.1% Offensive unit = 3.5–5.1% Delay = 2.1–2.6% Defensive converage = 2.2–3.1% Defensive balance = 3.3–3.0% Recovery balance = 1.4–1.9% Concetration = 1.8–2.1% Defensive unit = 3.7–4.0% Tactical attack actions in offensive midfield = 4.4–3.7% Tactical attack actions in defensive midfield = 6.4–7.7% Tactical defense actions in offensive midfield = 6.5–5.2% Tactical defense actions in defensive midfield = 5.7–5.1%	HRAvg HRpeak	TD D % at 0 to 7.2, 7.3 to 14.3, 14.4 to 21.5 km·h^-1^ Max speed Acc (> 2.0 m/s^2^) % D Acc (> 2 m/s^2^) Max Acc	None	Penetration, Offensive coverage, width and length (with and without ball), depth mobility, offensive unit, delay, defensive converage, defensive balance, recovery balance, concetration, defensive unit, tactical actions (in attack and defense)
[53]	6 vs 6 with 2 floating players Within session	N = 10 Age:28.9 ± 3.6 CL: P	IL: TRIMP [63]	None	93–100% HRmax = 5.16 mmol; 86–92% HRmax = 3.61 mmol; 79–85% HRmax = 2.54 mmol; 72–78% HRmax = 1.71 mmol; 65–71% HRmax = 1.25 mmol	None	None	None
[54]	5 vs 5 Within session Between session	N = 10 Age: 21.7 ± 2.1 CL: A	IL: RPE (CR-10 scale) HR (Polar H7, Polar Electro, OY, Kempele, Finland) EL: Geolocation tracker (JOHAN Sports, Noordwijk, The Netherlands) consisting of a GPS sensor (10 Hz, including EGNOS correction), accelerometer, gyroscope, and magnetometer (100 Hz, 3 axes)	None	RPE HRmean	TD D at 14–19.9 km·h^-1^, D > 20 km·h^-1^; TAcc (n/min); TDec (n/min); PL	None	None
[55]	1 vs 1 3 vs 3 Within session	N = 6 Age: 20.3 ± 4.8 CL: A	IL: RPE (CR-10 scale) HR – Polar Team app software with the use of the Polar H7 Bluetooth monitor (Polar Electro, OY, Kempele, Finland EL: Tracker (JOHAN Sports, Noordwijk, the Netherlands) consisting of a GPS sensor (10 Hz, including EGNOS correction), accelerometer, gyroscope, and magnetometer (100 Hz, 3 axes, ± 16 g). The GPS was tested with a 2.5 ± 0.41% (error ± deviation) reliability for TD	None	RPE HRAvg %HRmax	TD D at 0–6.9 7–13.9 14–19.9 > 20 km·h^-1^ PL Sprints nr Max speed Pace	None	none
[29]	5 vs 5 Within session Between session	N = 10 Age:18.3 ± 0.5 CL:A	EL: A 10-Hz GPS unit (including EGNOS correction, JOHAN Sports, Noordwijk, The Netherlands) and an accelerometer, gyroscope, and magnetometer (100 Hz, 3 axes, ± 16 g)	None	None	TD, D at 14–20 km·h^-1^ PL	None	None
[30]	GK + 5 vs 5 + GK Within session	N = 10 Age:28.1 ± 3.8 CL: P	EL: GPS at 10 Hz (OptimeEye S5, Catapult, Australia) equipped with an inertial measurement unit (100 Hz, 3 axes)	None	None	TD D > 14.4 19.8–25.1 > 25.1 km·h^-1^ Max speed; PL	None	None
[45]	3 vs 3 6 vs 6 Between session Within session	N = 16 Age:10.1 ± 0.3 CL: A	None	TE: Two planes (one at an open angle and the other focusing on the player with the ball) using two digital cameras (Go Pro Hero 2, 1280 × 960, 25 Hz)	None	None	RB CB LB AB S	None
[56]	3 vs 3 4 vs 4 5 vs 5 Within session	N = 16 Age:13.5 ± 0.7 CL:A	IL: HR (Polar Team System, Polar Electro Oy, Kempele, Finland)	TE: Video recordings of all SSGs were made with a camcorder (SC-D381/XAZ, Samsung Electronics America, Inc., Ridgefield Park, NJ, USA)	%HRmax	None	involvement with the ball, crosses, headers, tackles, shots on goal, dribbling, passing, and target passing	None
[34]	2 vs 2 3 vs 3 4 vs 4 Within session	N = 20 Age:27 ± 2 CL: P	IL: HR (Polar S-810, Polar-Electro OY, Kempele, Finland) Bl^-^ (Lactate Pro, Arkray, Japan) RPE scale (CR-10 scale) EL: GPSports SPI Elite System, Canberra, Australia) in which the distance travelled was recorded at 5 Hz	TE: 4 fixed digital video cameras	RPE %HRmax % HRres Bl^-^	TD D at 13–17 > 17 km·h^-1^	total nr of duels; successful passes; total nr of lost balls; total nr of ball possessions	None
[37]	4 vs 4 Within session	N = 20 Age:27.4 ± 1.5 CL: P	IL: RPE (CR-10 scale) Bl- (Lactate Pro, Arkray, Japan) HR (Polar S-810, Polar-Electro, Finland) EL: GPS (GPSports SPI Elite System, Canberra, Australia)	TE: four digital cameras	RPE Bl^-^ %HRmax %HRres	TD Distances at 13–17 km·h^-1^ and > 17 km·h^-1^	Nr of duels; % of successful passes; Nr of ball lost; Total nr of ball possession	None
[57]	2 vs 2 1 vs 1 Between session	N = 22 Age: 26.3 ± 4.7 CL: A	IL: HR (Polar RS800; Polar Electro, Kempele, Finland) RPE (CR-10 scale)	None	HRmax HRres RPE	None	None	None
[58]	3 vs 3 Within session	N = 19 Age: 24 ± 4 CL: A, P	IL: RPE (CR-10 scale) The reliability of heart rate (HR) during SSG range from 2.0 to 2.4% (typical error). HR – ND	TE: digital video camcorder (Canon MV700, miniDV, Canon Japan). Reliability of technical actions have been recently reported to be k = 0.82	RPE %HRmax	None	pass, successful pass, unsuccessful pass, tackle, header, turn, interception, dribbling, shoot, and shoot on target	None
[48]	GK + 4 vs 4 + GK Within session	N = 10 Age: 17.3 ± 0.7 CL: A	None	TB: local position measurement (LPM) system (Inmotio Object Tracking BV, Amsterdam, the Netherland)	None	None	None	The centroid and surface area relation between the teams
[16]	2 vs 2 4 vs 4 Within session	N = 16 Age: 16.3 ± 0.6 CL: A	IL: RPE (CR-20 scale) HR (Polar Team Sport System; Polar Electro, Kempele, Finland) Bl- – ND EL: GPS (SPI 10; GPSports, Canberra, Australia)	None	RPE %HRmax Blood lactate	TD D at 0 – 6.9 and > 18 km·h^-1^	None	None
[59]	2 vs 2 3 vs 3 4 vs 4 Within session	N = 14 Age: 16.7 ± 0.6 CL: A	IL: RPE (CR-10) Polar S810 HR (Polar Electro OY, Kempele, Finland) Bl- Plus analyzer (Nova Biomedical, Waltham, MA, USA)	None	RPE %HRmax Bl^-^	None	None	None
[35]	1 vs 1 2 vs 2 3 vs 3 4 vs 4 Within session	N = 16 Age: 15.7 ± 0.4 CL: A	IL: HR Polar S810 HR (Polar Electro OY, Kempele, Finland) Bl- analyzer (YSI Incorporated Life Sciences)	None	HR %HRmax Bl^-^	None	None	None
[46]	3 vs 3 Between session	N = 12 Age: 15 ± 3 CL: SM	None	TE: video recorder (Sony HDR-CX130), inter-observer reliability using Cohen’s Kappa (k > 0.814). Intra- class correlations showed a high degree of intra-observer reliability (ICC > 0.801)	None	None	Individual time in possession; individual touches in possession; team time in possession; successful team passes; % successful pass; intercept; deflection; unsuccessful pass; successful pass; Unsuccessful 1^st^ touch pass; Successful 1^st^ touch pass; Successful tackle; Unsuccessful tackle; Lost possession; Total possessions per bout; Technical actions per minute; Time ball is out of play	None
[36]	GK + 4 vs 4 + GK Between session	N = 10 Age: 20.2 ± 1.9 CL: A	IL: GPS (MinimaxX S4, Catapult Sports, Canberra, Australia). Typical error HRmax = 2.3 HR zones < 70 = 6.6 70–80 = 4.8 80–90 = 9.1 90–95 = 10.5 95–100% = 7.1 HRmean = 5.5 EL: GPS (MinimaxX S4, Catapult Sports, Canberra, Australia). Typical error TD = 229 Work rate = 5.7 PL (au) = 34 PL (m/min) = 0.8 Max speed = 1.4 0–2 = 15 | 2–5 = 60 5–7 = 46 | 7–9 = 75 9–13 = 124 | 13–16 = 41 16–20 = 43 | > 20 km/h = 24	None	HRmax HR zones < 70, 70–80, 80–90, 90–95, and 95–100% HRmax	TD, work rate, peak speed, nr of efforts (speed zone entries), and D at 0–2, 2–5, 5–7, 7–9, 9–13, 13–16, 16–20 and > 20 km·h^-1^ PL	None	None
[60]	3 vs 3 4 vs 4 5 vs 5 Within session	N = 12 Age: 12.8 ± 0.8 CL: A	EL: GPS SPI Elite (GPSports Systems, Pty. Ltd., 2003, Australia).	None	None	DT D at 0 – 4; 4.1 km·h^-1^ – MAV; MAV > MIV; D > MIV; Max speed.	None	None
[31]	7 vs 7 Within session	N = 14 Age: 20.9 ± 1.9 CL: A	EL: GPS (SP PRO X II GPSports®, 15 Hz, Canberra, Australia)	None	HRAvg	Avg D (m/min), and Avg speed (km·h^-1^); % D at 0–1, 11.1–14, 14.1–19 and 19.1–23 km·h^-1^	None	None
[61]	3 vs 3 Within session	N U12 = 12 U14 = 12 U16 = 12 Age: U12 = 11.8 ± 0.3 U14 = 12.8 ± 0.4 U16 = 15.3 ± 0.5 CL: A	IL: HR (Polar V800, Polar Electro, Finlandia) (Polar H7, Polar Electro, Finlandia) RPE (CR-10)	None	HRAvg %HRmax %HRres RPE	None	None	None
[62]	3 vs 3 GK + 3 vs 3 + GK GK + 4 vs 4 + GK 4 vs 4 GK + 6 vs 6 + GK 6 vs 6 Within session	N = 20 Age: 28.1 ± 4.6 CL: P	IL: HR (Polar H10, Polar-Electro, Kempele, Finland)	None	HRAvg, Edwards’TRIMP [64] Time spent in red zone (> 80% HRmax)	None	None	None
[21]	3 vs 3 GK + 3 vs 3 + GK GK + 4 vs 4 + GK 4 vs 4 GK + 6 vs 6 + GK 6 vs 6 Within session	N = 20 Age: 28.1 ± 4.6 CL: P	EL: GPS (VX Sport, Wellington, New Zealand)	None	None	TD, D at > 14.4; > 19.8 km·h^-1^; and MW; Acc/Dcc efforts (2.2 m·s^2^)	None	None

N: number of participants; IL: internal load; EL: external load; TE: technical execution; TB: tactical behavior; A: amateur; P: professional; SM: semi-professional; nr: number; GPS: global positioning system; D: distance; TD: total distance; TAcc: total acceleration; nr: number; TDec: total deceleration; PL: player load; MW: mechanical work; MAV: maximal aerobic velocity; MIV: maximal intermittent velocity; RPE: rated perceived exertion; HR: heart rate; Avg: average; HRres: heart rate reserve; HRmax: heart rate maximum; La-: lactate; red zone: > 80% of maximal HR; RB: received balls; CB: conquered balls; LB: lost balls; AB: attacking balls; S: shots; P: passes; TP: target passes; Avg: average.

The characteristics of the SSGs from the studies included in the systematic review can be found in table 4. There were three studies that applied 1 vs 1, five studies that applied 2 vs 2, 13 studies that applied 3 vs 3, 12 studies that applied 4 vs 4, four studies that applied 5 vs 5, four studies that applied 6 vs 6, one study that applied 7 vs 7 and one study that applied 4 vs 3 SSGs. Further characteristics regarding touch limitations, use of goalkeepers, pitch size, duration of work and rest intervals of the SSGs are also presented in table 4.

**TABLE 4:** Small-sided games characteristics

Study	Sessions tested (N)	Format of play	Pitch size (m)	RAP (m^2^)	Sets (N)	Sets (min)	Rest between sets (min)	Work-to-rest ratio*
[49]	1	3 vs 3 – free limitation	36 × 27	162	2	4	4	1/1
[49]	1	4 vs 3 – free limitation	36 × 27	138	2	4	4	1/1
[27]	1	4 vs 4 – free limitation	30 × 20	75	4	4	2	1/0.5
[53]	5	6 vs 6 + 2 (2 touch limitation)	30 × 20	42.9	3	8	2	1/0.25
[54]	6	5 vs 5 – free limitation	42 × 22	96.8	6	3	2	1/1.5
[54]	6	5 vs 5 – free limitation	42 × 22	96.8	3	6	2	1/0.33
[55]	1	1 vs 1 – free limitation	10 × 15	75	3	2	3	1/1.5
[55]	1	3 vs 3 – free limitation	19 × 24	76	3	3	9	1/1.5
[29]	2	5 vs 5 – free limitation	30 × 30	90	3	5	2	1/0.6
[30]	1	GK + 5 vs 5 + GK – 2 touch limitation	60 × 30	180	3	4	2	1/0.5
[45]	2	3 vs 3 – free limitation	15 × 20	50	3	3	2	1/0.67
[45]	2	6 vs 6 – free limitation	30 × 22	55	3	6	2	1/0.33
[56]	4	3 vs 3 – free limitation	30 × 30	150	3	4	3	1/0.75
[56]	4	4 vs 4 – free limitation	30 × 30	112.5	3	4	3	1/0.75
[56]	4	5 vs 5 – free limitation	30 × 30	90	3	4	3	1/0.75
[34]	1	2 vs 2 – 2 touch limitation	20 × 15	75	4	2	3	1/1.5
[34]	1	3 vs 3 – 2 touch limitation	25 × 18	75	4	3	3	1/1
[34]	1	4 vs 4 – 2 touch limitation	30 × 20	75	4	4	3	1/0.75
[37]	9	4 vs 4 – free limitation	30 × 20	75	4	4	3	1/0.75
[37]	9	4 vs 4 – 1 touch limitation	30 × 20	75	4	4	3	1/0.75
[37]	9	4 vs 4 – 2 touch limitation	30 × 20	75	4	4	3	1/0.75
[57]	9	2 vs 2 – free limitation	20 × 20	100	5	2.30	2	1/0.87
[57]	9	1 vs 1 – free limitation	15 × 10	75	5	1.30	1.5	1.30/1.5
[58]	1	3 vs 3 – 2 touch limitation	37 × 31	191.2	3	2	4	1/2
[58]	1	3 vs 3 – 2 touch limitation	37 × 31	191.2	3	4	4	1/2
[58]	1	3 vs 3 – 2 touch limitation	37 × 31	191.2	3	6	4	1/2
[48]	1	GK + 4 vs 4 + GK – free limitation	28 × 36	100.8	3	8	2	1/0.25
[16]	18	2 vs 2 – free limitation	28 × 21	147	6	4	1.5	1/0.37
[16]	18	4 vs 4 – free limitation	40 × 30	150	6	4	1.5	1/0.37
[59]	10	2 vs 2 – free limitation	12 × 24	72	8	2	2	1/1
[59]	10	3 vs 3 – free limitation	18 × 30	90	8	3	2	1/0.67
[59]	10	4 vs 4 – free limitation	24 × 36	108	8	4	2	4/2
[35]	1	1 vs 1 – free limitation	6 × 18	54	6	1	2	1/2
[35]	1	2 vs 2 – free limitation	12 × 24	288	6	2	2	1/1
[35]	1	3 vs 3 – free limitation	18 × 30	90	6	3	2	1/0.67
[35]	1	4 vs 4 – free limitation	24 × 36	108	6	4	2	1/0.5
[46]	8	3 vs 3 – free limitation	15 × 20	50	6	2	30	1/0.15
[46]	8	3 vs 3 – free limitation	15 × 20	50	6	2	120	1/1
[36]	2	GK + 4 vs 4 + GK	40 × 20	80	2	20	5	1/0.25
[60]	6	3 vs 3 – free limitation	14 × 22	51.32	3	4	1.50	1/0.37
[60]	6	4 vs 4 – free limitation	24 × 30	90	3	4	1.50	1/0.37
[60]	6	5 vs 5 – free limitation	30 × 48	144	3	4	1.50	1/0.37
[31]	2	7 vs 7 – free limitation	20 × 30	42.9	2	10	3	1/0.3
[31]	2	7 vs 7 – free limitation	30 × 40	42.9	2	10	3	1/0.3
[61]	12	3 vs 3 (U-12) – free limitation	20 × 25	83.2	4	4	3	1/0.75
[61]	12	3 vs 3 (U-14) – free limitation	20 × 25	83.2	4	4	3	1/0.75
[61]	12	3 vs 3 (U-16) – free limitation	20 × 25	83.2	4	4	3	1/0.75
[62]	9	3 vs 3 – free limitation	20 × 27	180	2	3	2	1/0.67
[62]	9	3 vs 3 -3 touch limitation	20 × 27	180	2	3	2	1/0.67
[62]	9	GK + 3 vs 3 + GK – free limitation	20 × 27	67.5	2	3	2	1/0.67
[62]	9	GK + 3 vs 3 + GK – 3 touch limitation	20 × 27	67.5	2	3	2	1/0.67
[62]	9	4 vs 4 – free limitation	22 × 32	88	2	4	2	1/0.5
[62]	9	4 vs 4 – 3 touch limitation	22 × 32	88	2	4	2	1/0.5
[62]	9	GK + 4 vs 4 + GK – free limitation	22 × 32	70.4	2	4	2	1/0.5
[62]	9	GK + 4 vs 4 + GK – 3 touch limitation	22 × 32	70.4	2	4	2	1/0.5
[62]	9	6 vs 6 – free limitation	28 × 40	93.2	2	6	2	1/0.33
[62]	9	6 vs 6 – 3 touch limitation	28 × 40	93.2	2	6	2	1/0.33
[62]	9	GK + 6 vs 6 + GK – free limitation	28 × 40	80	2	6	2	1/0.33
[62]	9	GK + 6 vs 6 + GK – 3 touch limitation	28 × 40	80	2	6	2	1/0.33
[21]	9	3 vs 3 – free touch	20 × 27	180	3	3	2	1/0.67
[21]	9	3 vs 3 – 3 touch limitation	20 × 27	180	3	3	2	1/0.67
[21]	9	4 vs 4 – free touch	22 × 32	88	3	4	2	1/0.5
[21]	9	4 vs 4 – 3 touch limitation	22 × 32	88	3	4	2	1/0.5
[21]	9	6 vs 6 – free touch	28 × 40	93.2	3	6	2	1/0.33
[21]	9	6 vs 6 – 3 touch limitation	28 × 40	93.2	3	6	2	1/0.33
[21]	9	GK + 3 vs 3 + GK – free touch	20 × 27	67.5	3	3	2	1/0.67
[21]	9	GK + 3 vs 3 + GK -3 touch limitation	20 × 27	67.5	3	3	2	1/0.67
[21]	9	GK + 4 vs 4 + GK – free touch	22 × 32	70.4	2	4	2	1/0.5
[21]	9	GK + 4 vs 4 + GK -3 touch limitation	22 × 32	70.4	2	4	2	1/0.5
[21]	9	GK + 6 vs 6 + GK – free touch	28 × 40	80	3	6	2	1/0.33
[21]	9	GK + 6 vs 6 + GK – 3 touch limitation	28 × 40	80	3	6	2	1/0.33

RAP: relative area per player area of the pitch divided by the number of players involved); GK: goalkeeper;

* : ratio expressed by minute.

### Results of individual studies: variability of internal load during SSGs

The synthesis of results can be found in table 5. There were 13 studies that analysed HR, six studies that analysed RPE, four studies that analysed lactate and one study that analysed TRIMP. In addition, there were six studies where was not possible to extract mean and standard deviation of the variables analyse, nine studies that did not present ICC or % CV and two studies where was not possible to extract any data.

**TABLE 5:** Quantitative synthesis for variability of IL outcomes in SSGs

Study	Format	Within-session (WS) and Between-session (BS) analysis	IL (ICC and %CV)	Significant or meaningful differences between sets/ repetitions (within-session WS and between-sessions BS)	Lowest and the highest sets/repetitions (within-session)	% of change between the lowest and the highest sets/ repetitions (within-session)
[49]	3 vs 3	WS: yes BS: not	ICC: HRAvg = 0.72 HRpeak = 0.51	WS: not BS: NA	ND	ND
[49]	4 vs 3	WS: yes BS: not	ICC: HRAvg = 0.87 HRpeak = 0.61	WS: not BS: NA	ND	ND
[27]	4 vs 4	WS: yes BS: not	ICC: ND %CV: ND	WS: yes BS: NA	RPE: 12.3 ± 1.5 – 14.5 ± 1.9 HRpeak: 169 ± 23.9 -174 ± 17.6 HRAvg: 157 ± 23.9 – 161 ± 19.4 %HRpeak: 87.0 ± 10.5 -88.9 ± 8.3 < 75% HRpeak: 19.6 ± 32.6 – 25.6 ± 34.5 75–84% HRpeak: 10.3 ± 8.7 -14.5 ± 10.4 85–89% HRpeak: 7.5 ± 7.8 – 10.8 ± 10.0 > 90% HRpeak: 56.0 ± 31.4 -57.0 ± 32.2	RPE = 17.9 HRpeak = 3.0 HRAvg = 2.5 %HRpeak = 2.2 < 75% HRpeak = 30.6 75–84% HRpeak = 40.8 85–89% HRpeak = 44.0 > 90% HRpeak = 1.8
[53]	6 vs 6 + 2	WS: yes BS: not	ICC: ND %CV: 12.35 ± 4.62%	WS: yes BS: ND	ND	TRIMP = 15.2
[54]	5 vs 5 (6 sets)	WS: yes BS: yes	ICC: ND %CV: ND	WS: yes BS: yes	RPE: 4.2 ± 1.5 – 6.1 ± 1.9 HRmean: 165.2 ± 12.4 – 171.6 ± 10.0	RPE = 45.2 HRmean = 3.9
[54]	5 vs 5 (3 sets)	WS: yes BS: not	ICC: ND %CV: ND	WS: yes BS: not	RPE: 5.0 ± 1.2 – 6.7 ± 1.6 HRmean: 168.8 ± 10.5 – 171.1 ± 10.9	RPE = 34 HRmean = 1.4
[55]	1 vs 1	WS: yes BS: not	ICC: ND %CV: ND	WS: yes BS: NA	HRAvg: 171.0 ± 15.0 – 177.3 ± 11.3 %HRmax: 92.548 ± 5.3 – 94.325 ± 4.7	HRAvg = 3.7 %HRmax = 1.9
[55]	3 vs 3	WS: yes BS: not	ICC: ND %CV: ND	WS: yes BS: NA	HR average: 172.3 ± 9.9 – 175.0 ± 7.6 %HRmax: 93.3 ± 3.4 – 94.9 ± 2.5	HRAvg = 1.6 %HRmax = 1.7
[56]	3 vs 3	WS: yes BS: not	ICC: ND %CV: Exercise intensity = 2.9	WS: yes BS: NA	ND*	*
[56]	4 vs 4	WS: yes BS: not	ICC: ND %CV: Exercise intensity = 3.4	WS: yes BS: NA	ND*	*
[56]	5 vs 5	WS: yes BS: not	ICC: ND %CV: Exercise intensity = 2.2	WS: yes BS: NA	ND*	*
[34]	2 vs 2	WS: yes BS: not	ICC: ND %CV: ND	WS: yes BS: NA	RPE: 6.7 ± 0.2 – 8.9 ± 0.1 %HRmax: 86.6 ± 0.6 – 93.4 ± 0.4 %HRres: 80.0 ± 3.2 – 95.1 ± 3.3 La-: 2.6 ± 0.0 – 4.6 ± 0.1	RPE = 32.8 %HRmax = 7.9 %HRres = 18.9 La- = 76.9
[34]	3 vs 3	WS: yes BS: not	ICC: ND %CV: ND	WS: yes BS: NA	RPE: 6.8 ± 0.7 – 8.9 ± 0.7 %HRmax: 86.9 ± 2.6 – 91.9 ± 3.0 %HRres: 79.8 ± 3.9 – 92.5 ± 3.8 La-: 2.8 ± 0.2 – 3.9 ± 0.2	RPE = 30.9 %HRmax = 5.8 %HRres = 15.9 La- = 2.3
[34]	4 vs 4	WS: yes BS: not	ICC: ND %CV: ND	WS: yes BS: NA	RPE: 6.9 ± 0.7 – 8.9 ± 0.8 %HRmax: 83.4 ± 2.8 – 87.9 ± 3.2 %HRres: 77.6 ± 4.3 – 88.5 ± 4.4 La-: 2.5 ± 0.2 – 3.2 ± 0.2	RPE = 29 %HRmax = 5.4 %HRres = 14 La- = 28
[37]	4 vs 4 free limitation	WS: yes BS: not	ICC: ND %CV: ND	WS: yes BS: NA	RPE: 6.3 ± 0.5 – 8.2 ± 0.9 La-: 2.4 ± 0.3 – 4.5 ± 0.3 %HRmax: 82.7 ± 2.6 – 86.8 ± 2.9 %HRres: 77.2 ± 3.0 – 82.6 ± 3.8	RPE = 30.2 La- = 87.5 %HRres = 0.1 %HRres = 7
[37]	4 vs 4 1 touch limitation	WS: yes BS: not	ICC: ND %CV: ND	WS: yes BS: NA	RPE: 6.8 ± 0.8 – 8.9 ± 0.8 La-: 2.5 ± 0.2 – 3.5 ± 0.5 %HRmax: 85.0 ± 2.3 – 90.4 ± 2.7 %HRres: 80.1 ± 3.0 – 87.0 ± 3.3	RPE = 30.9 La- = 40 %HRres = 6.4 %HRres = 8.6
[37]	4 vs 4 2 touch limitation	WS: yes BS: not	ICC: ND %CV: ND	WS: yes BS: NA	RPE: 6.7 ± 0.8 – 8.9 ± 0.5 La-: 2.5 ± 0.1 – 3.2 ± 0.3 %HRmax: 83.4 ± 2.8 – 89.7 ± 3.2 %HRres: 78.0 ± 4.3 – 83.8 ± 4.4	RPE = 32.8 La- = 28 %HRres = 7.6 %HRres = 7.4
[57]	2 vs 2	WS: not BS: yes	ICC: ND %CV: ND	WS: ND BS: ND	ND	NA
[57]	1 vs 1	WS: not BS: yes	ICC: ND %CV: ND	WS: not BS: ND	ND	NA
[58]	3 vs 3 2 min set	WS: yes BS: not	ICC: ND %CV: ND	WS: yes BS: NA	%HRmax: 82.2 ± 3.7 – 82.5 ± 4.0 RPE: 6.3 ± 1.4 – 7.2 ± 1.9	%HRmax = 0.4 RPE = 14.3
[58]	3 vs 3 4 min set	WS: yes BS: not	ICC: ND %CV: ND	WS: yes BS: NA	%HRmax: 85.5 ± 4.5 – 86.3 ± 3.2 RPE: 6.2 ± 1.4 – 7.3 ± 1.5	%HRmax = 0.9 RPE = 1.3
[58]	3 vs 3 6 min set	WS: yes BS: not	ICC: ND %CV: ND	WS: yes BS: NA	%HRmax: 85.2 ± 3.6 – 86.3 ± 3.3 RPE: 6.1 ± 1.6 – 7.5 ± 1.4	%HRmax = 23 RPE = 7.5
[16]	2 vs 2	WS: yes BS: not	ICC: ND %CV: ND	WS: yes BS: yes	ND	NA
[16]	4 vs 4	WS: yes BS: not	ICC: ND %CV: ND	WS: yes BS: yes	ND	NA
[59]	2 vs 2	WS: yes BS: not	ICC: ND %CV: %HRmax = 3.8 La- = 26.4 RPE = 14.3	WS: yes BS: NA	ND*	NA
[59]	3 vs 3	WS: yes BS: not	ICC: ND %CV: %HRmax = 2.6 La^-^ = 19.6 RPE = 11.4	WS: yes BS: NA	ND*	NA
[59]	4 vs 4	WS: yes BS: not	ICC: ND %CV: %HRmax = 2.7 La^-^ = 20.5 RPE = 11.6	WS: yes BS: NA	ND*	NA
[35]	1 vs 1	WS: yes BS: not	ICC: ND %CV: ND	WS: yes BS: NA	HR:159.6 ± 13.6 – 171.6 ± 7.5 %HRmax: 81.4 ± 6.3 – 87.6 ± 4.0 La^-^: 5.9 ± 1.4 – 11.0 ± 3.6	HR = 7.5 %HRmax = 7.6 La- = 86.4
[35]	2 vs 2	WS: yes BS: not	ICC: ND %CV: ND	WS: yes BS: NA	HR: 158.4 ± 14.8 – 178.5 ± 6.5 %HRmax: 80.9 ± 7.5 – 91.2 ± 3.5 La^-^: 6.3 ± 2.8 – 9.5 ± 2.4	HR = 12.7 %HRmax = 12.7 La- = 50.8
[35]	3 vs 3	WS: yes BS: not	ICC: ND %CV: ND	WS: yes BS: NA	HR: 177.0 ± 6.4 – 185.3 ± 6.6 %HRmax: 90.4 ± 3.0 – 94.6 ± 3.2 La-: 5.3 ± 1.4 – 8.4 ± 3.1	HR = 4.7 %HRmax = 4.6 La- = 58.5
[35]	4 vs 4	WS: yes BS: not	ICC: ND %CV: ND	WS: yes BS: NA	HR: 171.6 ± 7.4 – 182.4 ± 8.8 %HRmax: 87.6 ± 4.3 – 93.1 ± 3.6 La^-^: 5.0 ± 1.3 – 8.2 ± 3.2	HR = 6.3 %HRmax = 6.3 La- = 64
[36]	GK + 4 vs 4 + GK	WS: not BS: yes	ICC: HRmean = 0.74 HRpeak = 0.75 HR zones < 70 = -0.16; 70–80 = -0.01; 80–90 = 0.67; 90–95 = 0.19; 95–100% = 0.79 %CV: HRmean = 6.0 HRpeak = 4.2 HR zones < 70 = 81.7; 70–80 = 36.2; 80–90 = 38.3; 90–95 = 36.5; 95–100% = 128.4	WS: not BS: yes	ND	NA
[31]	7 vs 7 20 × 30	WS: yes BS: not	ICC: ND %CV: ND	WS: yes BS: NA	HRAvg: 163.3 ± 9.2 – 166 ± 10.2	HRAvg = 1.7
[31]	7 vs 7 30 × 40	WS: yes BS: not	ICC: ND %CV: ND	WS: yes BS: NA	HRAvg: 152.1 ± 11.3 – 155.1 ± 18.6	HRAvg = 2
[61]	3 vs 3 U12	WS: yes BS: not	ICC: ND %CV: HRmean = 7.99 %HRmax = 4.69 %HRres = 8.68 RPE = 9.79	WS: yes BS: NA	HRmean: 156.17 ± 13.65 – 174.88 ± 11.79 %HRmax: 77.31 ± 3.69 – 86.59 ± 4.41 %HRres: 63.98 ± 5.49 – 78.58 ± 7.51 RPE: 6.25 ± 1.08 – 7.88 ± 1.00	HRmean = 12 %HRmax = 12 %HRres = 22 RPE = 26.1
[61]	3 vs 3 U14	WS: yes BS: not	ICC: ND %CV: HRmean = 7.24 %HRmax = 5.75 %HRres = 12.35 RPE = 5.67	WS: yes BS: NA	HRmean: 159.58 ± 12.90 – 172.33 ± 11.54 %HRmax: 79.66 ± 4.55 – 86.01 ± 5.36 %HRres: 66.37 ± 8.69 – 76.60 ± 9.55 RPE: 7.13 ± 0.6 8.13 ± 0.71	HRmean = 8 %HRmax = 8 %HRres = 15.4 RPE = 14
[61]	3 vs 3 U16	WS: yes BS: not	ICC: ND %CV: HRmean = 8.05 %HRmax = 6.97 %HRres = 11.01 RPE = 8.94	WS: yes BS: NA	HRmean: 161.71 ± 12.12 – 173.42 ± 13.91 %HRmax: 80.82 ± 5.0 – 86.67 ± 6.00 %HRres: 70.44 ± 7.92 – 79.67 ± 8.86 RPE: 6.63 ± 1.07 – 7.71 ± 0.69	HRmean = 7.2 %HRmax = 7.2 %HRres = 13.1 RPE = 16.3
[62]	3 vs 3 – free limitation	WS: yes BS: not	ICC: HRAvg = 0.89 TRIMP = 0.67 Red zone = 0.71	WS: yes BS: NA	HRAvg: 157.1 ± 7.0 – 157.7 ± 7.5 TRIMP: 3.3 ± 0.2 – 3.4 ± 0.2 Red zone: 0.6 ± 0.1 – 0.6 ± 0.1	HRAvg = 0.4 TRIMP = 3 Red zone = 0
[62]	3 vs 3 - 3 touch limitation	WS: yes BS: not	ICC: HRAvg 0.94 TRIMP 0.82 Red zone 0.80 %CV: HRAvg 1.3 TRIMP 3.0 Red zone 8.2	WS: yes BS: NA	HRAvg: 156.4 ± 7.3 – 157.3 ± 7.7 TRIMP: 3.3 ± 0.2 – 3.3 ± 0.2 Red zone: 0.6 ± 0.1 – 0.6 ± 0.1	HRAvg = 0.6 TRIMP = 0 Red zone = 0
[62]	GK + 3 vs 3 + GK – free limitation	WS: yes BS: not	ICC: HRAvg 0.96 TRIMP 0.96 Red zone 0.96 %CV: HRAvg 1.0 TRIMP 2.2 Red zone 5.8	WS: yes BS: NA	HRAvg: 159.1 ± 8.0 – 159.6 ± 7.6 TRIMP: 3.6 ± 0.4 – 3.8 ± 0.4 Red zone: 0.7 ± 0.1 – 0.7 ± 0.2	HRAvg = 0.3 TRIMP = 5.6 Red zone = 0
[62]	GK + 3 vs 3 + GK – 3 touch	WS: yes BS: not	ICC: HRAvg 0.97 TRIMP 0.86 Red zone 0.92 %CV: HRAvg 1.0 TRIMP 3.7 Red zone 7.3	WS: yes BS: NA	HRAvg: 157.4 ± 8.3 – 158.8 ± 8.3 TRIMP: 3.6 ± 0.3 – 3.7 ± 0.3 Red zone: 0.7 ± 0.1 – 0.7 ± 0.2	HRAvg = 0.9 TRIMP = 2.8 Red zone = 0
[62]	GK + 4 vs 4 + GK Free limitation	WS: yes BS: not	ICC: HRAvg 0.65 TRIMP 0.66 Red zone 0.75 %CV: HRAvg 1.7 TRIMP 3.4 Red zone 8.5	WS: yes BS: NA	HRAvg: 160.3 ± 8.2 – 161.6 ± 5.7 TRIMP: 3.5 ± 0.3 – 3.6 ± 0.3 Red zone: 0.6 ± 0.1 – 0.6 ± 0.2	HRAvg = 0.8 TRIMP = 2.9 Red zone = 0
[62]	GK + 4 vs 4 + GK 3 touch limitation	WS: yes BS: not	ICC: HRAvg 0.78 TRIMP 0.86 Red zone 0.88 %CV: HRAvg 1.7 TRIMP 8.5 Red zone 3.4	WS: yes BS: NA	HRAvg: 160.8 ± 7.0 -161.4 ± 6.0 TRIMP: 3.5 ± 0.3 – 3.6 ± 0.3 Red zone: 0.6 ± 0.1 – 0.6 ± 0.1	HRAvg = 0.4 TRIMP = 2.9 Red zone = 0
[62]	4 vs 4 – free limitation	WS: yes BS: not	ICC: HRAvg 0.94 TRIMP 0.86 Red zone 0.91 %CV: HRAvg 1.2 TRIMP 2.8 Red zone 7.5	WS: yes BS: NA	HRAvg: 157.3 ± 8.2 – 158.1 ± 8.0 TRIMP: 0.6 ± 0.1 – 0.6 ± 0.1 Red zone: 3.3 ± 0.2 – 3.4 ± 0.2	HRAvg = 0.5 TRIMP = 0 Red zone = 3
[62]	4 vs 4 – 3 touch limitation	WS: yes BS: not	ICC: HRAvg 0.96 TRIMP 0.84 Red zone 0.96 %CV: HRAvg 0.9 TRIMP 3.2 Red zone 5.7	WS: yes BS: NA	HRAvg: 158.3 ± 7.6 – 158.5 ± 7.2 TRIMP: 3.5 ± 0.3 – 3.5 ± 0.3 Red zone: 0.6 ± 0.2 – 0.6 ± 0.1	HRAvg = 0.1 TRIMP = 0 Red zone = 0
[62]	GK + 6 vs 6 + GK – free limitation	WS: yes BS: not	ICC: HRAvg 0.93 TRIMP 0.92 Red zone 0.85 %CV: HRAvg 1.4 TRIMP 2.2 Red zone 8.4	WS: yes BS: NA	HRAvg: 159.4 ± 7.0 – 161.6 ± 7.6 TRIMP: 3.7 ± 0.4 – 3.6 ± 0.4 Red zone: 0.6 ± 0.2 – 0.7 ± 0.2	HRAvg = 1.4 TRIMP = 2.7 Red zone = 16.7
[62]	GK + 6 vs 6 + GK – 3 touch limitation	WS: yes BS: not	HRAvg 0.93 TRIMP 0.93 Red zone 0.97 %CV: HRAvg 1.0 TRIMP 2.0 Red zone 3.8	WS: yes BS: NA	HRAvg: 158.8 ± 7.4 – 159.5 ± 7.2 TRIMP: 3.6 ± 0.3 – 3.7 ± 0.3 Red zone: 0.6 ± 0.1 – 0.7 ± 0.1	HRAvg = 0.4 TRIMP = 2.8 Red zone = 16.7
[62]	6 vs 6 – free limitation	WS: yes BS: not	ICC: HRAvg 0.83 TRIMP 0.77 Red zone 0.78 %CV: HRAvg 1.7 TRIMP 4.9 Red zone 6.4	WS: yes BS: NA	HRAvg: 163.8 ± 7.1 – 164.1 ± 6.9 TRIMP: 3.8 ± 0.3 – 3.9 ± 0.3 Red zone: 0.7 ± 0.1 – 0.8 ± 0.1	HRAvg = 0.2 TRIMP = 2.6 Red zone = 14.3
[62]	6 vs 6 – 3 touch limitation	WS: yes BS: not	ICC: HRAvg 0.88 TRIMP 0.82 Red zone 0.92 %CV: HRAvg 1.6 TRIMP 3.0 Red zone 5.4	WS: yes BS: NA	HRAvg: 164.0 ± 6.6–164.4 ± 6.1 TRIMP: 3.9 ± 0.3 – 3.9 ± 0.3 Red zone: 0.7 ± 0.1 – 0.7 ± 0.1	HRAvg = 0.2 TRIMP = 0 Red zone = 0

IL: internal load; ICC: intra-class correlation; %CV: percentage of coefficient of variation; ND: non-described; NA: non-applicable. RPE: rated perceived exertion; HR: heart rate; Avg: average; HRres: heart rate reserve; HRmax: heart rate maximum; La^-^: lactate; red zone: > 80% of maximal HR;

* : non-extractable data.

### Results of individual studies: variability of external load during SSGs

The synthesis of results can be found in table 6. There were 11 studies that analysed distance covered variables, three studies that analysed acceleration, two studies that analysed deceleration, five studies that analysed player load, and one study that analysed mechanical work. There were three studies where was not possible to extract mean and standard deviation of the variables analysed, seven studies that did not present ICC or % CV and one study where was not possible to extract any data.

**TABLE 6:** Quantitative synthesis for variability of EL outcomes in SSGs

Study	Format	Within-session (WS) and Between-session (BS) analysis	EL (ICC and % CV)	Significant or meaningful differences between sets/ repetitions (within-session WS and between-sessions BS)	lowest and the highest sets/ repetitions (within-session)	% of change between the lowest and the highest sets/repetitions (within-session)
[49]	3 vs 3	WS: yes BS: not	ICC: TD = 0.68 D% 0–7.2 km = 0.38 D% 7.3–14.3 km = 0.56 D% 14.4–21.5 km = 0.54 Max speed = 0.08 Acc nr (> 2 m/s) = 0.66 % D Acc (> 2 m/s) = 0.51 Acc max = -0.29 %CV = ND	WS: not BS: NA	ND	ND
[49]	4 vs 3	WS: yes BS: not	ICC: TD = 0.71 D% 0–7.2 km = 0.42 D% 7.3–14.3 km = 0.74 D% 14.4–21.5 km = 0.28 Max speed = 0.09 Acc nr (> 2 m/s) = 0.24 % D Acc (> 2 m/s) = 0.27 Max Acc = 0.24 %CV = ND	WS: not BS: NA	ND	ND
[27]	4 vs 4	WS: yes BS: not	ICC: ND %CV: ND	WS: yes BS: NA	TD: 500 ± 53.4 – 533 ± 48.3 D at 0–6.9 km/h: 179 ± 20.9 – 200 ± 28.0 D at 7.0–12.9 km/h: 249 ± 52.1 – 289 ± 45.2 D at 13–17.9 km/h: 49.9 ± 27.5 – 67.5 ± 33.3 D ≥ 18.0 km/h: 1.8 ± 3.0 – 6.5 ± 6.6 Total m/min: 125 ± 13.4 – 133 ± 11.9 TAcc nr: 231 ± 10.7 – 233 ± 12.2 Acc (1–1.4 m·min^-2^): 36.3 ± 8.6 – 46.8 ± 4.6 Acc (1.5–1.9 m·min^-2^): 30.8 ± 9.2 – 39.1 ± 6.4 Acc (2–2.4 m·min^-2^): 25.7 ± 6.4 – 28.5 ± 5.3 Acc (≥ 2.5 m·min^-2^): 119 ± 9.6 – 138 ± 19.1 TDec nr: 228 ± 14.3 – 232 ± 15.2 Max speed: 18.0 ± 1.5 – 19.9 ± 2.7 Avg speed: 6.5 ± .6 – 7.0 ± .4	TD = 6.6 D at 0–6.9 km/h = 11.7 D at 7.0–12.9 km/h = 16.1 D at 13–17.9 km/h = 35.3 D ≥ 18.0 km/h = 261.1 Total m/min = 6.4 TAcc nr = 0.9 Acc (1–1.4 m·min^-2^) = 28.9 Acc (1.5–1.9 m·min^-2^) = 26.9 Acc (2–2.4 m·min^-2^) = 10.9 Acc (≥ 2.5 m·min^-2^) = 16 TDec nr = 1.8 Max speed = 10.6 Avg speed = 7.7
[54]	5 vs 5 (6 set)	WS: yes BS: yes	ICC: ND %CV: ND	WS: yes BS: yes	TD: 101.6 ± 10.9 -112.5 ± 11.1 D at 14–19.9 km/h: 9.1 ± 6.6 – 14.1 ± 5.7 D 20 km/h: 0.3 ± 0.7 – 1.5 ± 1.9 TAcc: 2.2 ± 1.0 – 2.9 ± 0.8 TDec: 1.7 ± 0.9 – 2.7 ± 0.9 PL: 6.4 ± 1.1 – 7.3 ± 1.3	TD = 10.7 D at 14–19.9 km/h = 54.9 D 20 km/h = 400 TAcc = 31.8 TDec = 58.8 PL = 14.1
[54]	5 vs 5 (3 set)	WS: yes BS: no	ICC: ND %CV: ND	WS: yes BS: no	TD: 90.9 ± 15.6 – 103.3 ± 7.6 D at 14–19.9 km/h: 6.0 ± 3.8 – 9.4 ± 5.6 D 20 km/h: 0.5 ± 0.8 – 0.7 ± 1.3 TAcc: 1.8 ± 0.9 – 2.1 ± 1.0 TDec: 1.6 ± 0.9 – 1.9 ± 1.0 PL: 5.9 ± 1.3 – 6.9 ± 0.9	TD = 13.6 D at 14–19.9 km/h = 56.7 D 20 km/h = 40 TAcc = 16.7 TDec = 18.8 PL = 16.9
[55]	1 vs 1	WS: yes BS: not	ICC: ND %CV: ND	WS: yes BS: NA	TD: 218.8 ± 22.1 – 240.4 ± 15.4 D at 0–6.9 km/h: 91.0 ± 6.0 – 102.1 ± 10.7 D at 7–13.9 km/h: 102.2 ± 25.2 – 127.1 ± 13.0 D at 14–19.9 km/h: 14.5 ± 9.2 – 22.1 ± 18.4 D 20 km/h: 0.2 ± 0.5 – 0.4 ± 0.8 PL (volume): 15.2 ± 2.3 – 16.6 ± 1.6 Nr of sprints: 0.2 ± 0.4 – 0.3 ± 0.5 Max speed: 17.8 ± 2.4 – 18.9 ± 1.4 Pace: 110.3 ± 11.1 – 119.2 ± 7.6 PL intensity: 7.8 ± 1.1 – 8.2 ± 0.8	TD = 9.9 D at 0–6.9 km/h = 12.2 D at 7–13.9 km/h = 24.4 D at 14–19.9 km/h = 52.4 D 20 km/h = 100 PL (volume) = 9.2 Nr of sprints = 50 Max speed = 6.2 Pace = 8.1 PL intensity = 5.1
[55]	3 vs 3	WS: yes BS: not	ICC: ND %CV: ND	WS: yes BS: NA	TD: 420.0 ± 55.6 – 456.2 ± 51.2 D at 0–6.9 km/h: 189.3 ± 22.5 – 202.1 ± 21.6 D at 7–13.9 km/h: 169.0 ± 54.7 – 205.2 ± 61.2 D at 14–19.9 km/h: 37.1 ± 23.8 – 59.5 ± 23.2 D 20 km/h: 1.0 ± 0.9 – 3.4 ± 5.9 PL (volume): 24.8 ± 5.3 – 27.7 ± 4.7 Nr of sprints: 0.8 ± 0.8 – 1.0 ± 1.6 Max speed: 19.8 ± 1.7 – 21.2 ± 3.1 Pace: 105.0 ± 13.9 – 114.0 ± 12.8 PL intensity: 6.2 ± 1.3 – 7.0 ± 1.2	TD = 8.6 D at 0–6.9 km/h = 6.8 D at 7–13.9 km/h = 21.4 D at 14–19.9 km/h = 60.4 D 20 km/h = 240 PL (volume) = 11.7 Nr of sprints = 25 Max speed = 7.1 Pace = 8.6 PL intensity = 12.9
[29]	5 vs 5	WS: yes BS: yes	ICC: ND %CV: TD = 6.9; 8.3% D at 14–20 km/h = 53.3; 145.7%, PL = 4.9; 6.0%,	WS: yes BS: ND	ND*	NA
[30]	GK + 5 vs 5 + GK	WS: yes BS: not	ICC: ND %CV: TD = 8.64% D 14.4 km/h = 45.96% Max speed = 12.31% D at 19.8–25.1 km/h = 64.30% D 19.8 km/h = 68.66% PL = 5.23%	WS: yes BS: NA	TD: 103.8 ± 8.8 – 122.4 ± 7.4 D 14.4 km/h: 9.6 ± 4.9 – 27.0 ± 10.9 Max speed: 19.7 ± 2.6 – 24.8 ± 2.9 D at 19.8–25.1 km/h: 1.0 ± 1.7 – 5.6 ± 4.0 D 19.8 km/h: 1.0 ± 1.7 – 7.0 ± 5.3 PL: 11.5 ± 1.1 -12.7 ± 1.2	TD = 17.9 D 14.4 km/h = 181.3 Max speed = 25.9 D at 19.8–25.1 km/h = 460 D 19.8 km/h = 600 PL = 10.4
[34]	2 vs 2	WS: yes BS: not	ICC: ND %CV: ND	WS: yes BS: NA	TD: 273.0 ± 30.8 – 332.3 ± 22.1 D 17 km/h: 36.7 ± 6.5 – 58.9 ± 5.3 D at 13–17 km/h: 54.9 ± 11.6 – 78.5 ± 9.1	TD = 21.7 D 17 km/h = 60.5 D at 13–17 km/h = 43.0
[34]	3 vs 3	WS: yes BS: not	ICC: ND %CV: ND	WS: yes BS: NA	TD: 461.8 ± 30.3 – 584.4 ± 56.2 D 17 km/h: 72.6 ± 12.9 – 100.2 ± 12.3 D at 13–17 km/h: 99.7 ± 6.9 – 144.9 ± 20.9	TD = 26.5 D 17 km/h = 38 D at 13–17 km/h = 45.3
[34]	4 vs 4	WS: yes BS: not	ICC: ND %CV: ND	WS: yes BS: NA	TD: 604.9 ± 55.2 – 711.9 ± 65.5 D 17 km/h: 76.5 ± 13.4 – 103.6 ± 14.6 D at 13–17 km/h: 107.7 ± 13.2 – 169.8 ± 20.5	TD = 17.7 D 17 km/h = 35.4 D at 13–17 km/h = 57.7
[37]	4 vs 4 free limitation	WS: yes BS: not	ICC: ND %CV: ND	WS: yes BS: NA	TD: 597.6 ± 56.7 – 726.3 ± 65.4 D 17 km/h: 80.9 ± 13.4 – 107.3 ± 15.6 D at 13–17 km/h: 101.3 ± 12.1 – 142.3 ± 25.7	TD = 21.5 D 17 km/h = 32.6 D at 13–17 km/h = 40.5
[37]	4 vs 4 – 1 touch limitation	WS: yes BS: not	ICC: ND %CV: ND	WS: yes BS: NA	TD: 668.7 ± 73.9 – 835.7 ± 61.1 D 17 km/h: 102.1 ± 12.6 – 140.7 ± 20.4 D at 13–17 km/h: 132.0 ± 16.6 – 195.7 ± 14.9	TD = 25 D 17 km/h = 37.8 D at 13–17 km/h = 48.3
[37]	4 vs 4 – 2 touch limitation	WS: yes BS: not	ICC: ND %CV: ND	WS: yes BS: NA	TD: 604.9 ± 55.2 – 711.9 ± 65.5 D 17 km/h: 76.5 ± 13.4 – 103.6 ± 14.6 D at 13–17 km/h: 107.7 ± 13.2 – 169.8 ± 20.5	TD = 17.7 D 17 km/h = 35.4 D at 13–17 km/h = 57.7
[16]	2 vs 2	WS: yes BS: not	ICC: ND %CV: ND	WS: yes BS: NA	ND	NA
[16]	4 vs 4	WS: yes BS: not	ICC: ND %CV: ND	WS: yes BS: NA	ND	NA
[36]	GK + 4 vs 4 + GK	WS: not BS: yes	ICC: TD = 0.39 Work rate = 0.39 PL (au) = 0.54 PL (m/min) = 0.54 Max speed = 0.63 D at 0–2 = 0.72 2–5 = 0.59 | 5–7 = 0.74 | 7–9 = 0.12 | 9–13 = -0.09 | 13–16 = 0.75 | 16–20 = 0.57 | 20 km/h = 0.74 CV: TD = 7.8 Work rate = 7.9 PL (au) = 12.9 PL (m/min) = 12.6 Max speed = 8.5 D at 0–2 = 18.4 2–5 = 8.5 | 5–7 = 12.9 | 7–9 = 18.2 | 9–13 = 15.7 | 13–16 = 22.1 | 16–20 = 25.7 20 km/h = 47.6	WS: not BS: yes	ND	NA
[60]	3 vs 3	WS: yes BS: not	ICC: ND %CV: ND	WS: yes BS: NA	DT: 288.1 ± 46.6 – 333.2 ± 63.9 D at 0–4 km/h: 90.8 ± 14.8 – 94.3 ± 17.8 D at 4.1 km/h – MAV: 166.9 ± 53.3 -183.8 ± 36.3 MAV MIV: 25.7 ± 9.6 – 46.5 ± 40.2 D MIV: 3.7 ± 5.8 – 9.9 ± 1.5 Max speed: 14.2 ± 0.9 – 15.2 ± 1.5	DT = 15.7 D at 0–4 km/h = 3.9 4.1 km/h – MAV = 10.1 MAV MIV = 80.9 D MIV = 167.6 Max speed = 7
[60]	4 vs 4	WS: yes BS: not	ICC: ND %CV: ND	WS: yes BS: NA	DT: 356.7 ± 46.6 – 373.2 ± 46.9 D at 0–4 km/h 86.9 ± 14.8 – 90.5 ± 16.5 D at 4.1 km/h – MAV: 209.4 ± 51.0 – 220.9 ± 41.8 MAV MIV: 43.7 ± 19.6 – 55.5 ± 22.4 D MIV: 10.6 ± 9.7 – 18.4 ± 9.7 Max speed: 16.1 ± 2.1 – 17.1 ± 1.7	DT = 4.6 D at 0–4 km/h = 4.1 4.1 km/h – MAV = 5.5 MAV MIV = 27 D MIV = 73.6 Max speed = 6.2
[60]	5 vs 5	WS: yes BS: not	ICC: ND %CV: ND	WS: yes BS: NA	DT: 393.3 ± 39.9 – 422.6 ± 49.1 D at 0–4 km/h 76.4 ± 16.9 – 94.3 ± 14.7 D at 4.1 km/h – MAV: 210.0 ± 36.0 – 232.2 ± 42.1 MAV MIV: 64.6 ± 26.4 – 78.2 ± 20.5 D MIV: 24.4 ± 15.0 – 33.7 ± 16.8 Max speed: 16.7 ± 1.9 – 18.9 ± 1.6	DT = 7.4 D at 0–4 km/h = 23.4 4.1 km/h – MAV = 10.6 MAV MIV = 21.1 D MIV = 38.1 Max speed = 13.2
[31]	7 vs 7 -free limitation	WS: yes BS: not	ICC: ND %CV: Cumulative max speed = 1.7% Max speed = 1.2%	WS: yes BS: NA	TD: 105.9 ± 9 – 107.4 ± 8.3 Avg Speed: 6.4 ± 0.5 – 6.4 ± 0.5 D at 0–11 km/h: 826.5 ± 56.5 – 848 ± 47.5 D at 11.1–14 lm/h: 152.1 ± 38.5 –154.6 ± 32.9 D at 14.1–19 km/h: 70.4 ± 28.7 – 75.6 ± 14.6 D at 19.1–23 km/h: 3.9 ± 5 – 9.7 ± 9.2	TD = 1.4 Avg Speed = 0 D at 0–11 km/h = 2.6 D at 11.1–14 lm/h = 1.6 D at 14.1–19 km/h = 7.4 D at 19.1–23 km/h = 148.7
[31]	7 vs 7 – free limitation	WS: yes BS: not	ICC: ND %CV: Cumulative max speed = 1.7% Max speed = 1.2%	WS: yes BS: NA	TD: 115.7 ± 7.4 – 116.3 ± 10.5 Avg Speed: 6.9 ± 0.4 – 7 ± 0.6 D at 0–11 km/h: 816.2 ± 65.3 – 848 ± 47.5 D at 11.1–14 lm/h: 193.6 ± 50.1 – 198 ± 24.6 D at 14.1–19 km/h: 121.4 ± 50.2 -121.9 ± 42.4 D at 19.1–23 km/h: 17.9 ± 14.4 – 23.9 ± 25.9	TD = 0.5 Avg Speed = 1.4 D at 0–11 km/h = 3.9 D at 11.1–14 lm/h = 2.3 D at 14.1–19 km/h = 0.4 D at 19.1–23 km = 33.5
[21]	3 vs 3 – free touch	WS: yes BS: not	ICC: TD = 0.80 D 14.4 km/h = 0.92 D 19.8 km/h = 0.49 MW = 0.48 %CV: TD = 4.3 D 14.4 km/h = 9.3 D 19.8 km/h = 21.4 MW = 9.4	WS: yes BS: NA	TD: 127.1 ± 11.4 – 134.1 ± 14.5 D 14.4 km/h: 14.0 ± 4.2 – 14.9 ± 3.6 D 19.8 km/h: 1.6 ± 0.4 – 1.9 ± 0.5 MW: 4.0 ± 0.5 – 4.1 ± 0.6	TD = 5.5 D 14.4 km/h = 6.4 D 19.8 km/h = 18.8 MW = 2.5
[21]	3 vs 3 – 3 touch limitation	WS: yes BS: not	ICC: TD = 0.74 D 14.4 km/h = 0.89 D 19.8 km/h = 0.51 MW = 0.47 %CV: TD = 4.1 D 14.4 km/h = 10.3 D 19.8 km/h = 17.7 MW = 9.8	WS: yes BS: NA	TD: 127.4 ± 11.3 – 129.8 ± 8.6 D 14.4 km/h: 14.5 ± 3.7 – 15.0 ± 4.0 D 19.8 km/h: 1.7 ± 0.4 – 2.0 ± 0.5 MW: 4.1 ± 0.5 – 4.5 ± 0.5	TD = 1.9 D 14.4 km/h = 3.4 D 19.8 km/h = 17.6 MW = 9.8
[21]	4 vs 4 – free touch	WS: yes BS: not	ICC: TD = 0.67 D 14.4 km/h = 0.78 D 19.8 km/h = 0.60 MW = 0.61 %CV: TD = 4.6 D 14.4 km/h = 11.6 D 19.8 km/h = 14.2 MW = 10.2	WS: yes BS: NA	TD: 127.4 ± 11.3 – 129.8 ± 8.6 D 14.4 km/h: 14.5 ± 3.7 – 15.0 ± 4.0 D 19.8 km/h: 1.7 ± 0.4 – 2.0 ± 0.5 MW: 4.4 ± 0.6 – 5.2 ± 0.9	TD = 1.9 D 14.4 km/h = 3.4 D 19.8 km/h = 17.6 MW = 18.2
[21]	4 vs 4 – 3 touch limitation	WS: yes BS: not	ICC: TD = 0.83 D 14.4 km/h = 0.90 D 19.8 km/h = 0.79 MW = 0.74 %CV: TD = 3.7 D 14.4 km/h = 8.8 D 19.8 km/h = 12.2 MW = 12.4	WS: yes BS: NA	TD: 115.1 ± 9.5 – 118.1 ± 10.4 D 14.4 km/h: 16.8 ± 3.9 – 17.7 ± 3.8 D 19.8 km/h: 2.3 ± 0.5 – 2.7 ± 0.7 MW: 6.8 ± 1.7 – 6.9 ± 1.3	TD = 2.6 D 14.4 km/h = 5.4 D 19.8 km/h = 17.4 MW = 1.5
[21]	6 vs 6 – free touch	WS: yes BS: not	ICC: TD = 0.92 D 14.4 km/h = 0.92 D 19.8 km/h = 0.48 MW = 0.79 %CV: TD = 2.3 D 14.4 km/h = 10.5 D 19.8 km/h = 29.4 MW = 17.0	WS: yes BS: NA	TD: 117.0 ± 9.6 – 118.3 ± 8.7 D 14.4 km/h: 10.3 ± 3.4 – 10.8 ± 3.8 D 19.8 km/h: 1.7 ± 0.5 – 1.9 ± 0.5 MW: 6.1 ± 2.0 – 6.6 ± 2.5	TD = 1.1 D 14.4 km/h = 4.9 D 19.8 km/h = 11.8 MW = 8.2
[21]	6 vs 6 – 3 touch limitation	WS: yes BS: not	ICC: TD = 0.87 D 14.4 km/h = 0.84 D 19.8 km/h = 0.91 MW = 0.83 %CV: TD = 2.7 D 14.4 km/h = 11.1 D 19.8 km/h = 17.3 MW = 11.8	WS: yes BS: NA	TD: 107.7 ± 8.7 – 108.3 ± 8.9 D 14.4 km/h: 9.9 ± 2.6 – 10.3 ± 2.6 D 19.8 km/h: 1.7 ± 0.7 – 1.8 ± 0.7 MW: 6.3 ± 1.7 – 6.6 ± 1.9	TD = 0.6 D 14.4 km/h = 4 D 19.8 km/h = 5.9 MW = 4.8
[21]	GK + 3 vs 3 + GK – free touch	WS: yes BS: not	ICC: TD = 0.92 D 14.4 km/h = 0.88 D 19.8 km/h = 0.79 MW = 0.84 %CV: TD = 3.1 D 14.4 km/h = 13.9 D 19.8 km/h = 18.2 MW = 10.6	WS: yes BS: NA	TD: 125.5 ± 14.9 – 127.0 ± 14.3 D 14.4 km/h: 18.5 ± 6.6 – 20.3 ± 6.3 D 19.8 km/h: 2.1 ± 0.5 – 2.6 ± 0.7 MW: 5.2 ± 1.3 – 5.4 ± 1.5	TD = 1.2 D 14.4 km/h = 9.7 D 19.8 km/h = 23.8 MW = 3.8
[21]	GK + 3 vs 3 + GK -3 touch limitation	WS: yes BS: not	ICC: TD = 0.95 D 14.4 km/h = 0.96 D 19.8 km/h = 0.81 MW = 0.71 %CV: TD = 2.9 D 14.4 km/h = 8.8 D 19.8 km/h = 16.0 MW = 16.2	WS: yes BS: NA	TD: 115.3 ± 14.8 – 121.2 ± 15.4 D 14.4 km/h: 16.2 ± 7.6 – 16.9 ± 8.4 D 19.8 km/h: 1.6 ± 0.6 – 2.7 ± 0.9 MW: 4.6 ± 1.5 – 5.9 ± 2.4	TD = 5.1 D 14.4 km/h = 4.3 D 19.8 km/h = 68.8 MW = 28.3
[21]	GK + 4 vs 4 + GK – free touch	WS: yes BS: not	ICC: TD = 0.85 D 14.4 km/h = 0.58 D 19.8 km/h = 0.76 MW = 0.56 %CV: TD = 2.7 D 14.4 km/h = 16.4 D 19.8 km/h = 19.0 MW = 22.5	WS: yes BS: NA	TD: 128.4 ± 9.5 – 135.1 ± 7.3 D 14.4 km/h: 15.3 ± 3.6 – 18.5 ± 3.7 D 19.8 km/h: 2.5 ± 0.8 – 3.2 ± 0.8 MW: 6.2 ± 2.1 – 6.5 ± 1.5	TD = 5.2 D 14.4 km/h = 20.9 D 19.8 km/h = 28 MW = 4.8
[21]	GK + 4 vs 4 + GK -3 touch limitation	WS: yes BS: not	ICC: TD = 0.92 D 14.4 km/h = 0.90 D 19.8 km/h = 0.75 MW = 0.87 %CV: TD = 2.9 D 14.4 km/h = 7.2 D 19.8 km/h = 16.7 MW = 14.7	WS: yes BS: NA	TD: 122.5 ± 12.7 – 126.1 ± 10.0 D 14.4 km/h: 17.0 ± 3.5 – 17.8 ± 3.9 D 19.8 km/h: 2.5 ± 0.8 – 2.6 ± 0.9 MW: 6.8 ± 3.0 – 7.3 ± 3.3	TD = 2.9 D 14.4 km/h = 4.7 D 19.8 km/h = 4 MW = 7.4
[21]	GK + 6 vs 6 + GK – free touch	WS: yes BS: not	ICC: TD = 0.84 D 14.4 km/h = 0.76 D 19.8 km/h = 0.77 MW = 0.72 %CV: TD = 3.2 D 14.4 km/h = 15.9 D 19.8 km/h = 26.3 MW = 18.4	WS: yes BS: NA	TD: 115.4 ± 8.7 – 116.4 ± 8.7 D 14.4 km/h: 11.8 ± 4.4 – 12.8 ± 2.9 D 19.8 km/h: 2.2 ± 1.3 – 2.4 ± 1.3 MW: 5.0 ± 1.8 – 5.9 ± 1.8	TD = 0.9 D 14.4 km/h = 8.5 D 19.8 km/h = 9.1 MW = 18
[21]	GK + 6 vs 6 + GK – 3 touch limitation	WS: yes BS: not	ICC: TD = 0.93 D 14.4 km/h = 0.92 D 19.8 km/h = 0.94 MW = 0.81 %CV: TD = 2.1 D 14.4 km/h = 7.8 D 19.8 km/h = 16.1 MW = 14.3	WS: yes BS: NA	TD: 111.0 ± 8.8 – 112.0 ± 8.8 D 14.4 km/h: 13.6 ± 3.8 – 13.9 ± 3.3 D 19.8 km/h: 3.3 ± 2.3 – 3.4 ± 2.1 MW: 5.8 ± 2.0 – 6.6 ± 1.9	TD = 0.9 D 14.4 km/h = 2.2 D 19.8 km/h = 3 MW = 3.8

EL: external load; ICC: intra-class correlation; %CV: percentage of coefficient of variation; D: distance; TD: total distance; TAcc: total acceleration; nr: number; TDec: total deceleration; PL: player load; MW: mechanical work; MAV: maximal aerobic velocity; MIV: maximal intermittent velocity;

* : non-extractable data.

### Results of individual studies: variability of technical execution during SSGs

The synthesis of results can be found in table 7. From the six studies, there were two with the inclusion of ICC or % CV, mean and standard deviation of the technical execution variables analysed in simultanesously, only one with the inclusion of ICC or % CV without including other information, two studies with mean and standard deviation of the technical execution variables, and 1 study that was not possible to extract any data.

**TABLE 7:** Quantitative synthesis for variability of TE outcomes in SSGs

Study	Format	Within-session (WS) and Between-session (BS) analysis	TE (ICC and % CV)	Significant or meaningful differences between sets/ repetitions (within-session WS and between-sessions BS)	Lowest and the highest sets/repetitions (within-session)	% of change between the lowest and the highest sets/repetitions (within-session)
[45]	3 vs 3	WS: yes BS: yes	ICC: not %CV: RB = 26.4 CB = 107.5 LB = 50.6 AB = 79.6 S = 62.2	WS: yes BS: not	RB: 3.5 ± 1.8 – 5.0 ± 2.3 CB: 1.9 ± 1.2 – 2.2 ± 1.9 LB: 1.7 ± 0.7 – 2.0 ± 0.8 AB: 1.3 ± 0.5 – 2.0 ± 1.5 S: 1.6 ± 0.7 – 2.5 ± 1.1	RB = 42.9 CB = 15.8 LB = 17.6 AB = 53.8 S = 56.3
[45]	6 vs 6	WS: yes BS: yes	ICC: not %CV: RB = 52.2 CB = 133.8 LB = 80.1 AB = 90.1 S = 40.6	WS: yes BS: not	RB: 2.9 ± 1.3 – 3.7 ± 1.9 CB: 1.3 ± 0.7 – 2.2 ± 1.5 LB: 1.1 ± 0.3 – 1.9 ± 1.6 AB: 1.1 ± 0.3 – 2.1 ± 1.4 S: 1.0 ± 0.0 – 1.5 ± 0.8	RB = 27.6 CB = 69.2 LB = 72.7 AB = 90.9 S = 50
[56]	3 vs 3	WS: yes BS: not	ICC: not %CV: Involvement with the ball = 8.5 P = 16.1 TP = 19.3	WS: yes BS: NA	ND*	NA
[56]	4 vs 4	WS: yes BS: not	ICC: not %CV: Involvement with the ball = 10.4 P = 15.2 TP = 16.7	WS: yes BS: NA	ND*	NA
[56]	5 vs 5	WS: yes BS: not	ICC: not %CV: Involvement with the ball = 8.3 P = 6.8 TP = 8.4	WS: yes BS: NA	ND*	NA
[34]	2 vs 2	WS: yes BS: not	ICC: not %CV: not	WS: yes BS: NA	Total nr of duels: 4.2 ± 1.2 – 8.7 ± 1.4 P: 55.5 ± 4.1 – 64.2 ± 4.9 LB: 2.4 ± 0.6 – 5.1 ± 1.2 Total nr of ball possessions: 10.4 ± 0.3 – 10.7 ± 0.3	Total nr of duels = 107.1 P = 15.7 LB = 112.5 Total nr of ball possessions = 2.9
[34]	3 vs 3	WS: yes BS: not	ICC: not %CV: not	WS: yes BS: NA	Total nr of duels: 4.7 ± 1.1 – 8.4 ± 1.0 P: 65.8 ± 2.2 – 72.4 ± 2.2 LB: 2.6 ± 0.9 – 5.4 ± 1.5 Total nr of ball possessions: 8.3 ± 1.2 – 11.2 ± 1.2	Total nr of duels = 78.7 P = 10 LB = 107.7 Total nr of ball possessions = 34.9
[34]	4 vs 4	WS: yes BS: not	ICC: not %CV: not	WS: yes BS: NA	Total nr of duels: 3.1 ± 0.8 – 5.7 ± 1.1 P: 63.5 ± 5.6 – 70.8 ± 5.1 LB: 2.6 ± 0.9 – 6.0 ± 1.7 Total nr of ball possessions: 8.3 ± 1.2 – 8.7 ± 1.6	Total nr of duels = 83.9 P = 11.5 LB = 130.8 Total nr of ball possessions = 4.8
[37]	4 vs 4 – free limitation	WS: yes BS: not	ICC: not %CV: not	WS: yes BS: NA	Nr of Duels: 4.1 ± 0.9 – 7.7 ± 1.2 P: 69.9 ± 7.8 – 75.9 ± 6.7 LB: 2.4 ± 1.2 – 4.4 ± 1.5 Total nr of ball possession: 7.3 ± 1.4 – 8.3 ± 2.1	Nr of Duels = 87.8 P = 8.6 LB = 83.3 Total nr of ball possession = 13.7
[37]	4 vs 4 – 1 touch limitation	WS: yes BS: not	ICC: not %CV: not	WS: yes BS: NA	Nr of Duels: 3.3 ± 0.9 – 5.3 ± 1.1 P: 44.7 ± 5.6 – 53.1 ± 5.3 LB: 2.5 ± 0.9 – 5.7 ± 1.9 Total nr of ball possession: 9.1 ± 2.8 – 12.6 ± 2.1	Nr of Duels = 60.6 P = 18.8 LB = 128 Total nr of ball possession = 38.5
[37]	4 vs 4 – 2 touch limitation	WS: yes BS: not	ICC: not %CV: not	WS: yes BS: NA	Nr of Duels: 3.0 ± 0.8 – 5.7 ± 1.1 P: 63.7 ± 5.6 – 70.8 ± 5.1 LB: 2.6 ± 0.9 – 4.2 ± 1.2 Total nr of ball possession: 8.5 ± 2.1 – 8.9 ± 2.0	Nr of Duels = 90 P = 11.1 LB = 61.5 Total nr of ball possession = 4.7
[58]	3 vs 3	WS: yes BS: not	ICC: not %CV: not	WS: yes BS: NA	ND*	NA*
[46]	3 vs 3 (30 rest)	WS: yes BS: not	ICC > 0.801 %CV: not	WS: yes BS: NA	Time in possession: 1.9 ± 0.8 – 2.7 ± 3.2 Touches in possession: 2.8 ± 0.5 – 4.1 ± 2.3 Avg team possession: 6.7 ± 2.0 – 9.8 ± 6.9 Pass/possession (team): 1.6 ± 0.3 – 3.2 ± 1.4 P: 79 ± 20 – 85 ± 12 Interceptions: 0.2 ± 0.4 – 0.4 ± 0.7 Deflections: 0.0 ± 0.0 – 0.3 ± 0.5 Unsuccessful pass: 0.4 ± 0.5 – 0.9 ± 0.8 Successful pass: 2.8 ± 1.5 – 4.0 ± 1.7 Unsuccessful 1^st^ touch pass: 0.1 ± 0.3 – 0.5 ± 0.8 Successful 1^st^ touch pass: 1.1 ± 0.9 – 2.0 ± 1.0 Successful tackle: 0.3 ± 0.5 – 0.9 ± 0.8 Unsuccessful tackle: 0.3 ± 0.5 – 0.9 ± 0.9 Technical actions: 3.3 ± 1.3 – 4.0 ± 1.3 LB: 0.2 ± 0.4 – 0.7 ± 0.9 Total possession per bout: 5.4 ± 1.6 – 7.5 ± 2.5	Time in possession = 42.1 Touches in possession = 46.4 Avg team possession = 46.3 Pass/possession (team) = 100 P = 7.6 Interceptions = 100 Deflections = 300 Unsuccessful pass = 125 Successful pass = 42.9 Unsuccessful 1^st^ touch pass = 400 Successful 1^st^ touch pass = 81.8 Successful tackle = 200 Unsuccessful tackle = 200 Technical actions = 21.2 LB = 250 Total possession per bout = 38.9
[46]	3 vs 3 (120 rest)	WS: yes BS: not	ICC > 0.801 %CV: not	WS: yes BS: NA	Time in possession: 1.8 ± 0.8 – 2.3 ± 1.2 Touches in possession: 3.0 ± 08 – 3.6 ± 1.5 Avg team possession: 5.9 ± 2.0 – 7.0 ± 2.7 Pass/possession: 1.4 ± 0.3 – 3.5 ± 3.4 P: 71 ± 29 – 81 ± 18 Interceptions: 0.1 ± 0.3 – 0.8 ± 0.8 Deflections: 0.0 ± 0.0 – 0.5 ± 1.2 Unsuccessful pass: 0.7 ± 0.7 – 1.3 ± 1.9 Unsuccessful 1^st^ touch pass: 0.3 ± 0.5 – 0.7 ± 0.5 Successful 1^st^ touch pass: 1.2 ± 0.9 – 1.7 ± 1.3 Successful tackle: 0.5 ± 0.7 – 1.3 ± 1.2 Unsuccessful tackle: 0.3 ± 0.5 – 0.9 ± 1.0 Technical actions: 3.4 ± 1.1 – 4.1 ± 1.4 LB: 0.3 ± 0.5 – 1.0 ± 0.9 Total possession per bout: 6.3 ± 1.9 – 7.3 ± 2.5	Time in possession = 27.8 Touches in possession = 20 Avg team possession = 18.6 Pass/possession (team) = 150 P = 14.1 Interceptions = 700 Deflections = 500 Unsuccessful pass = 85.7 Unsuccessful 1^st^ touch pass = 41.7 Successful 1^st^ touch pass = 160 Successful tackle = 200 Unsuccessful tackle = 20.6 Technical actions LB = 233.3 Total possession per bout = 15.9

TE: technical execution; ICC: intra-class correlation; %CV: percentage of coefficient of variation; ND: non-described; NA: non-applicable. RB: received balls; CB: conquered balls; LB: lost balls; AB: attacking balls; S: shots; P: passes; TP: target passes; Avg: average;.

* : non-extractable data.

### Results of individual studies: variability of tactical behavior during SSGs

The synthesis of results of the two studies that include behavior variables can be found in table 8. One study only presented ICC for all tactical behavior variables analysed while the other did present any extractable data.

**TABLE 8:** Quantitative synthesis for variability of TB outcomes in SSGs

Study	Format	Within-session (WS) and Between-session (BS) analysis	TB (ICC and % CV)	Significant or meaningful differences between sets/ repetitions (within-session WS and between-sessions BS)	Lowest and the highest sets/repetitions (within-session)	% of change between the lowest and the highest sets/repetitions (within-session)
[49]	3 vs 3	WS: yes BS: not	ICC: Penetration = 0.07 Offensive coverage = 0.40 Width and length with ball = 0.17 Width and length without ball = 0.06 Depth mobility = 0.35 Offensive unit = 0.40 Delay = 0.01 Defensive converage = 0.36 Defensive balance = 0.02 Recovery balance = 0.35 Concetration = 0.078 Defensive unit = 0.22 Tactical attack actions in offensive midfield = 0.38 Tactical attack actions in defensive midfield = 0.36 Tactical defense actions in offensive midfield = 0.16 Tactical defense actions in defensive midfield = 0.07 %CV: ND	WS: not BS: NA	ND	ND
[49]	4 vs 3	WS: yes BS: not	ICC: Penetration = 0.14 Offensive coverage = 0.16 Width and length with ball = 0.21 Width and length without ball = 0.43 Depth mobility = 0.40 Offensive unit = 0.23 Delay = -0.13 Defensive converage = 0.19 Defensive balance = 0.01 Recovery balance = 0.17 Concetration = 0.07 Defensive unit = 0.20 Tactical attack actions in offensive midfield = 0.69 Tactical attack actions in defensive midfield = 0.23 Tactical defense actions in offensive midfield = 0.34 Tactical defense actions in defensive midfield = 0.27 %CV: ND	WS: not BS: NA	ND	ND
[48]	GK + 4 vs 4 + GK	WS: yes BS: not	ICC: ND TB: ND	WS: not BS: not	ND*	NA*

TB: tactical behavior; ICC: intra-class correlation; %CV: percentage of coefficient of variation; ND: non-described;

* : non-extractable data.

## DISCUSSION

Based on a systematic review of the available literature, this study aimed to identify studies that have examined the intra- and inter SSG bouts/set variability levels of internal and external load and technical/tactical outcomes in soccer players. Internal load and low-speed external load variables presented low variability, while high variation was reported for technical execution and high-speed external loads. However, tactical behavior variability was assessed by only two studies. The main topics of this review are further addressed in the following sections.

### Variability of internal load during SSGs

In the current systematic review, large within-session variability was observed for the RPE and the time ≤ 89% of the HRpeak (~15–44% of change between the lowest and highest sets/repetitions) [27]. In contrast, the %HRAvg, %HRpeak, %HRmax showed small withinsession variations (~0.5–6% of change between the lowest and highest sets/repetitions) [21, 27–32]. Perceived efforts are expected that increase across the sets/repetitions within sessions, especially when intra-player responses are analyzed. However, the possible variability of IL between teammates during the same SSGs (depending on the positional role and contextual factors, among other aspects) should be carefully analyzed by coaches so that they can properly compensate the training with more analytical tasks [5].

On the one hand, an analysis of La^-^ concentration in professional players showed a smaller format (e.g., 3 vs. 3) resulted in small within-session variability (~2% of change between the lowest and highest sets/repetitions) compared to a medium format (e.g., 4 vs. 4) (~28–87% of change between the lowest and highest sets/repetitions) [33, 34]. On the other hand, the 1 vs. 1 format showed greater within-session variability in La^-^ concentration (~86% of change between the lowest and highest sets/repetitions) than the 2 vs. 2 format (~51%), 3 vs. 3 (~58%), and 4 vs. 4 (~64%) in young amateur players [35]. The divergence of the results can be attributed to the competitive level (professional vs. amateur) and age group (senior vs. young players).

Only two studies included in the present review investigated the between-session variability of IL indicators. Nine SSG sessions (1 vs. 1 and 2 vs. 2) showed mean RPE values between ~7–8 AU (CR-10 scale) in amateur male adult players [34]. However, the authors did not show the statistical values for between-session variability (e.g., CV%, TEM). In recreational soccer SSGs, the percentage of time in each HR zone showed large between-session variability (CV = 36.2–128.4%). In contrast, minimal between-session variability was noted for HRmean and HRpeak, with CV values of 3.4% and 2.6%, respectively [36]. However, the current findings on this topic are limited, as only two studies have met all the inclusion criteria. Therefore, further studies should investigate the betweensession variability of IL, particularly in youth and professional elitelevel soccer players.

### Variability of external load during SSGs

The within-session variability of EL during SSGs was independent of task constraints (e.g., free touch limitations vs. one, two, or three-touch limitations), age-group (e.g., amateur vs. professional vs. youth level), and format (e.g., 1 vs. 1, 2 vs. 2, 3 vs. 3, 4 vs. 4, 5 vs. 5, 7 vs. 7). Absolute (m) and relative TD (m/min) presented small-to-moderate within-session variability during SSGs (%CV = ~5–9; ~1–10% of change between the lowest and highest sets/repetitions) [21, 27–31]. In contrast, large within-session variability (%CV = 45–146; ~35–400% of change between the lowest and highest sets/repetitions) was noted, mainly for high-speed efforts [21, 27–31, 34, 37]. These results suggest that highly demanding running speeds are highly variable within-session, and this could be compensated by planning training sessions with more analytical tasks [5].

Events related to changes in speed (e.g., number of acceleration/decelerations, distance covered in high accelerations/decelerations) presented inconsistent values (small to large) of within-session variability (ICC = 0.54; %CV = ~0.5–6; ~2.5–59% of change between the lowest and highest sets/repetitions) [21, 27–30, 36]. However, in general, lower within-session variations were observed for the mechanical load derived from inertial sensors/accelerometers (e.g., player load) compared to distances covered in high-speed zones (e.g., distance > 19.8 km·h^-1^) and events related to changes in speed (e.g., accelerations/decelerations).

Similar to the previous discussion on IL variability, only two studies investigated the between-session variability of EL during SSGs. Moderate-to-large between-session variations were observed for TD (%CV = ~7–8) and high-speed distance (%CV = ~16–146) in U-19 players [29] and in healthy untrained young adult players [36]. In contrast, player load showed small variations (%CV = ~5–6) [29]. In addition, a previous umbrella review reported that running demands during SSGs are highly dependent on tactical issues (e.g., rules, coaches’ intervention/encouragement, scoring line) [38]. Therefore, using mechanical load derived from the inertial sensors/accelerometers seems to be the most stable analysis method and might be the best way to monitor fitness and fatigue during SSGs [39].

SSGs, as well as official soccer matches, can be considered dynamic systems that involve relationships between two teams under the influence of different positional and contextual factors [5]. Therefore, all training scenarios involve some level of unpredictability, which naturally leads to an increase in the variability of stimuli [16]. This variability is essential for developing the tactical and technical aspects of the game and, in turn, solving problems that emerge during SSGs (see the discussion about within- and between-session variations of technical-tactical outcomes). However, considerable variability between teammates and sessions may not be ideal for developing physiological and physical traits. A more controlled variability level might be better when considering that the training load should be logically progressed, individualized, and standardized [40].

### Variability of technical execution during SSGs

Regarding technical execution, considerable within-session variation was reported in the selected studies, while no study has investigated between-session variations, which signifies a gap in the literature. From an ecological perspective, motor responses arise due to the emerging problems in a given task [41]. For this reason, when adopting SSGs, it seems plausible to expect that players’ responses will be highly variable, as they can adapt their behavior to create novel contexts. For example, a recent study showed that players’ behavioral efficiency was higher in the last bout than in the first [42], which supports the rationale that different technical executions are likely to be observed over successive bouts (within-session variation). Also, the task constraints seem to indicate players’ preferable methods for solving the emerging problems [43, 44]. At this point, the majority of the analyzed studies have included free tasks with no specific constraints, which might have increased the variation as a large number of different motor responses were possible. For this reason, we suggest that future studies test whether including more restrictive task constraints reduces the variability of technical execution in SSGs.

Another possible explanation for the large variation in the technical actions performed by players might be related to the characteristics of the measures. Specifically, technical actions are low-frequency discrete variables—as such, changing one single unity from one bout to the next could significantly increase within-session variation. For example, a previous study found that the frequencies of technical execution were always lower than 5 AU [45], which explains why the CV values were higher than 40% for every variable. In that study, even for the most frequent variable (rebounds), for which the highest value was 5 AU, one single additional execution would increase the total frequency by 20%. On the other hand, variables with higher frequencies might present lower CV values. In another study, the % of change between the lowest and highest sets/repetitions (within-session) was 10.0% for passes (frequencies ranged from 65.8 ± 2.2 – 72.4 ± 2.2), while the reported % of change for lost balls was 107.7% (frequencies ranged from 2.6 ± 0.9 – 5.4 ± 1.5) [34]. Also, in the study of McLean et al. (2016), the ICC values were higher than 0.801 for all investigated variables. In this study, some variables were continuously measured (e.g., time in possession and average team possession), while the other two were unities with decimal values (touches per possession and passes per possession), which might justify the higher ICC reported in comparison to other studies.

For these reasons, we argue that within-session variations in aspects related to technical execution might be higher for low-frequency discrete variables. Further investigations should be conducted on more reliable variables to describe technical execution in SSGs.

Higher variability naturally reduces prescription quality, as the coach is unlikely to determine the exact stimuli being experienced by the players. At this point, we recommend exercising caution when designing SSGs to promote specific technical actions, especially in high-performance contexts. On the other hand, it has been proposed that increasing variability is required to nurture players’ creativity, especially in the early stages of deliberate practice sessions [47]. For this reason, the large variability in technical execution should not be seen as an inherently negative aspect of SSG training but instead as a characteristic that might be considered when prescribing different task conditions.

### Variability of tactical behavior during SSGs

The high variability of tactical behaviors during SSGs is expected considering the previously mentioned rationale regarding the unpredictability of the actions in game-based scenarios. This feature of SSGs might not be seen as inherently negative, either, as it might nurture players’ creativity [47], as has also been mentioned. However, the current findings are limited in terms of eliciting a discuss this topic, as only one study met all the inclusion criteria.

Only one study [48] evaluated the within and between-session tactical variability in SSGs. Specifically, this study found no within- or between-session differences in tactical behavior measured by positional data. This result is different from those of a previous study that reported mainly weak ICC values for the within-session reliability of the frequency of tactical actions (core tactical principles) [49]. We argue that the difference between those studies is related to the characteristics of the variables as was previously introduced regarding the technical execution. Specifically, even if high behavioral variability could be expected in game-based scenarios (like SSGs), we argue that this variability will be enhanced when analyzing discrete low-frequency variables.

In the study of Bredt et al. [49], the assessed data corresponded to the tactical principles performed by each player. Meanwhile, Frencken et al. [50] collected positional data at 45 Hz using a local positioning system, which significantly increased the sample used for analysis. However, since few studies included data on the reliability and variability of tactical behavior in SSGs, we recommend further investigation on this topic. Specifically, future studies testing different task constraints could include data regarding the ICC or the CV of the variables to allow the reader to understand the expected variability of each task condition.

4.5. Study limitations, future research, and practical implications Few studies have investigated the between-session variability of internal load (n = 2), external load (n = 2), technical outcomes (n = 0), and tactical outcomes (n = 1) during soccer SSGs, particularly in young and professional elite-level players. Furthers studies should fill this gap in the literature. In addition, future studies should test whether including more restrictive task constraints reduces the variability of internal/external load and technical/tactical outcomes during SSGs. Regarding the methodological quality assessment, ~40% of the included studies presented a low level, which might represent a methodological limitation of the included results.

Coaches should consider three main practical implications when planning SSGs: i) %HRAvg, %HRpeak, %HRmax (more stable within sessions), and RPE scores (more stable between sessions) seem to be the best IL indicators; ii) mechanical load derived from the inertial sensors/accelerometers seems to be the most stable level of analysis and may have greater potential for monitoring fitness and fatigue; and iii) large variability in technical/tactical outcomes should not be seen as an inherently negative aspect of the training process with SSGs but as a characteristic that might be considered when prescribing different task conditions. Possibly, the dynamic of the games and some specific conditions as pitch size or goal-setting can play a determinant role to modulate the variability of the high-demanding match running and technical skills, mainly in cases in which few frequencies of events occur and in which standard deviation may cause a considerable impact on the variability.

## CONCLUSIONS

The current systematic review allowed to identify that some of the measures related to SSG responses can be more or less variable and this should be carefully understood by the coaches. In summary, it was found that internal load and low-speed external load variables presented low variability between repetitions and sessions for the same format, while high variations were reported for technical execution and high-speed external loads. Therefore, the use of SSGs should be planned based on the type of exposure selected by the coach. Eventually, for cardiorespiratory-based stimulus, the SSGs can be interesting since they present stable and low-variable stimulus in terms of internal load demands. However, for promoting mechanical stimulus while performing high-intensity runs, eventually, SSG can be too heterogenous and variable within and between-players, and maybe running-based exercises should be more recommended [51, 52]. Therefore, it is important to highlight such variability levels, at least to recommend a stronger monitoring process to control the dose imposed and adjust based on the player’s needs.

## Conflicts of interest

All the authors declare that they have no conflicts of interest relevant to the content of this review.

## Funding

Filipe Manuel Clemente: This work is funded by Fundação para a Ciência e Tecnologia/Ministério da Ciência, Tecnologia e Ensino Superior through national funds and when applicable co-funded EU funds under the project UIDB/EEA/50008/2020. Hugo Sarmento gratefully acknowledge the support of a Spanish government subproject Integration ways between qualitative and quantitative data, multiple case development, and synthesis review as main axis for an innovative future in physical activity and sports research [PGC2018-098742-B-C31] (Ministerio de Economía y Competitividad, Programa Estatal de Generación de Conocimiento y Fortalecimiento Científico y Tecnológico del Sistema I + D + i), that is part of the coordinated project ‘New approach of research in physical activity and sport from mixed methods perspective (NARPAS_MM) [SPGC201800X098742CV0]’. No other specific sources of funding were used to assist in the preparation of this article.

## Authorship Contributions

FMC lead the project, run the data search and methodological assessment, and wrote and revised the original manuscript. RA, GMP, RO, AFS and HS wrote and revised the original manuscript. MRG and JA run the data search and methodological assessment and wrote and revised the original manuscript.
